# Supplementary material for: One-year clinical outcomes of patients with versus without acute coronary syndrome with 3-month duration of dual antiplatelet therapy after everolimus-eluting stent implantation
Source: PLoS One. 2020 Mar 25;15(3):e0227612. doi: 10.1371/journal.pone.0227612 (PMC7094877; doi:10.1371/journal.pone.0227612)
Supplement: S1 Table — (DOCX) [file pone.0227612.s001.docx]

**S1 Table. Patient Characteristics: Enrolled versus Non-enrolled Patients**

|  | Enrolled | Non-enrolled | P Value |
| --- | --- | --- | --- |
|  | N=1525 | N=2054 |  |
| Age –years | 70.0±10.6 | 70.0±11.0 | 0.97 |
| Age >=75 years | 570 (37%) | 776 (38%) | 0.81 |
| Male gender | 1117 (73%) | 1553 (76%) | 0.11 |
| Body mass index | 24.1±3.6 | 23.9±3.6 (2010) | 0.04 |
| Coexisting condition |  |  |  |
| Hypertension | 1260 (83%) | 1574 (77%) | <0.0001 |
| Diabetes mellitus | 604 (40%) | 824 (40%) | 0.76 |
| Insulin-treated diabetes | 119 (7.8%) | 176 (8.6%) | 0.41 |
| ESRD (eGFR<30 mL/min/1.73m^2^) not on hemodialysis | 35/1521 (2.3%) | 93/2054 (4.5%) | 0.0003 |
| Hemodialysis | 56 (3.7%) | 141 (6.9%) | <0.0001 |
| Cardiac risk factor |  |  |  |
| Current smoker | 315 (21%) | 430 (21%) | 0.84 |
| Prior Stroke | 168 (11%) | 243 (12%) | 0.45 |
| Heart failure | 101 (6.6%) | 191 (9.3%) | 0.004 |
| Peripheral vascular disease | 142 (9.3%) | 177 (8.6%) | 0.47 |
| Clinical characteristics |  |  |  |
| Clinical presentation |  |  |  |
| Stable coronary artery disease | 1040 (68%) | 1277 (62%) | 0.0002 |
| Acute coronary syndrome | 485 (32%) | 777 (38%) | 0.0002 |
| Unstable angina | 229 (15%) | 299 (15%) | 0.7 |
| Acute myocardial infarction | 256 (17%) | 478 (23%) | <0.0001 |
| STEMI | 213 (14%) | 389 (19%) | <0.0001 |
| NSTEMI | 43 (2.8%) | 89 (4.3%) | 0.02 |
| Target-vessel location |  |  |  |
| Left main coronary artery | 17 (1.1%) | 160 (7.8%) | <0.0001 |
| Left anterior descending coronary artery | 866 (57%) | 1108 (54%) | 0.09 |
| Left circumflex coronary artery | 361 (24%) | 460 (22%) | 0.37 |
| Right coronary artery | 405 (27%) | 614 (30%) | 0.03 |
| Bypass graft | 4 (0.3%) | 17 (0.8%) | 0.02 |
| Complexity of coronary artery disease |  |  |  |
| Number of treated lesions per patient | 1.21±0.48 | 1.43±0.74 | <0.0001 |
| Multi-vessel treatment | 130 (8.5%) | 315 (15%) | <0.0001 |

Values are expressed as mean ± SD or number (%).

ESRD=end stage renal disease; eGFR=estimated glomerular filtration rate; STEMI=ST-segment elevation myocardial infarction; NSTEMI=non-ST-segment elevation myocardial infarction.
