# Supplementary material for: One-year clinical outcomes of patients with versus without acute coronary syndrome with 3-month duration of dual antiplatelet therapy after everolimus-eluting stent implantation
Source: PLoS One. 2020 Mar 25;15(3):e0227612. doi: 10.1371/journal.pone.0227612 (PMC7094877; doi:10.1371/journal.pone.0227612)
Supplement: S2 Table — (DOCX) [file pone.0227612.s002.docx]

**S2 Table. Patient Characteristics in Propensity Matched Cohort**

|  | ACS | Stable CAD | P Value |
| --- | --- | --- | --- |
|  | 487 patients | 487 patients |  |
| **Clinical characteristics** |  |  |  |
| Age – years | 69.0±12.3 | 69.9±10.1 | 0.22 |
| Age >=75 years * | 176 (36%) | 173 (36%) | 0.84 |
| Men | 361 (74%) | 352 (72%) | 0.51 |
| Body mass index | 24.0±3.6 | 24.3±3.5 | 0.13 |
| Hypertension | 406 (83%) | 389 (80%) | 0.16 |
| Diabetes mellitus * | 162 (33%) | 165 (34%) | 0.84 |
| Dyslipidemia | 386 (79%) | 420 (86%) | 0.004 |
| Hemodialysis * | 8 (1.6%) | 8 (1.6%) | 1.0 |
| Atrial fibrillation * | 39 (8.0%) | 39 (8.0%) | 1.0 |
| Anemia (Hemoglobin <11.0 g/dL) | 76 (16%) | 57 (12%) | 0.08 |
| Current smoker | 142 (29%) | 77 (16%) | <0.0001 |
| Prior myocardial infarction * | 38 (7.8%) | 37 (7.6%) | 0.9 |
| Prior Stroke | 49 (10%) | 44 (9.0%) | 0.57 |
| Heart failure | 58 (12%) | 45 (9.2%) | 0.18 |
| Peripheral vascular disease * | 19 (3.9%) | 20 (4.1%) | 0.87 |
| Prior percutaneous coronary intervention | 80 (16%) | 152 (31%) | <0.0001 |
| Multivessel disease | 141 (29%) | 197 (40%) | 0.0002 |
| Medications |  |  |  |
| Aspirin | 487 (100%) | 487 (100%) | - |
| Thienopyridines | 487 (100%) | 486 (99.8%) | 0.24 |
| Statins * | 438 (90%) | 439 (90%) | 0.91 |
| B-blockers | 256 (53%) | 152 (31%) | <0.0001 |
| ACE-I/ARB | 334 (69%) | 264 (54%) | 0.0001 |
| Anti-coaglants | 45 (9.2%) | 38 (7.8%) | 0.42 |
| **Lesion and procedural characteristics** |  |  |  |
| No. of treated lesions per patient | 1.14±0.42 | 1.26±0.56 | <0.0001 |
| No. of stents used per patient | 1.27±0.56 | 1.4±0.67 | 0.0008 |
| Total stent length per patient - mm | 30.7±18.0 | 33.2±21.2 | 0.048 |

Values are expressed as mean ± SD or number (%).

ACS=acute coronary syndrome; CAD=coronary artery disease; STOPDAPT=ShorT and OPtimal duration of Dual AntiPlatelet Therapy after everolimus-eluting cobalt-chromium stent; RESET=Randomized Evaluation of Sirolimus-eluting versus Everolimus-eluting stent Trial;, ESRD=end stage renal disease; eGFR=estimated glomerular filtration rate; STEMI=ST-segment elevation myocardial infarction; NSTEMI=non-ST-segment elevation myocardial infarction; ACE-I=angiotensin converting enzyme inhibitors; ARB=angiotensin II receptor blockers.

* Risk-adjusting variables selected for propensity matched analysis.
