# Supplementary material for: One-year clinical outcomes of patients with versus without acute coronary syndrome with 3-month duration of dual antiplatelet therapy after everolimus-eluting stent implantation
Source: PLoS One. 2020 Mar 25;15(3):e0227612. doi: 10.1371/journal.pone.0227612 (PMC7094877; doi:10.1371/journal.pone.0227612)
Supplement: S3 Table — (DOCX) [file pone.0227612.s003.docx]

**S3 Table. Clinical Outcomes at 12 months in Propensity Matched Cohort**

|  | No. of patients with at least one event | | P Value |
| --- | --- | --- | --- |
|  | (Cumulative incidence) | |  |
|  | ACS | Stable CAD |  |
|  | N=487 | N=487 |  |
| Primary Endpoint | 11 (2.3%) | 10 (2.1%) | 0.82 |
| Death |  |  |  |
| All-cause | 5 (1.0%) | 5 (1.0%) | 0.99 |
| Cardiovascular death | 2 (0.4%) | 3 (0.6%) | 0.66 |
| Myocardial infarction | 1 (0.2%) | 2 (0.4%) | 0.56 |
| Stroke | 4 (0.8%) | 3 (0.6%) | 0.7 |
| Bleeding |  |  |  |
| TIMI minor/major | 6 (1.2%) | 2 (0.4%) | 0.15 |
| GUSTO moderate/severe | 4 (0.8%) | 2 (0.4%) | 0.41 |
| Stent thrombosis |  |  |  |
| Possible | 0 (0%) | 2 (0.4%) | 0.16 |
| Probable | 0 (0%) | 0 (0%) |  |
| Definite | 0 (0%) | 0 (0%) |  |
| Cardiovascular death, MI, stroke or definite ST | 7 (1.4%) | 8 (1.7%) | 0.8 |
| Target-lesion revascularization | 8 (1.7%) | 6 (1.2%) | 0.58 |
| Target-vessel revascularization | 15 (3.1%) | 14 (2.9%) | 0.85 |
| Any coronary revascularization | 30 (6.2%) | 29 (6.0%) | 0.9 |

Values are expressed as number (%).

ACS=acute coronary syndrome; CAD=coronary artery disease; STOPDAPT=ShorT and OPtimal duration of Dual AntiPlatelet Therapy after everolimus-eluting cobalt-chromium stent; RESET=Randomized Evaluation of Sirolimus-eluting versus Everolimus-eluting stent Trial; TIMI=Thrombolysis in Myocardial Infarction; GUSTO=Global Utilization of Streptokinase and Tissue plasminogen activator for Occluded coronary arteries; MI=myocardial infarction; ST=stent thrombosis.
