# Supplementary material for: One-year clinical outcomes of patients with versus without acute coronary syndrome with 3-month duration of dual antiplatelet therapy after everolimus-eluting stent implantation
Source: PLoS One. 2020 Mar 25;15(3):e0227612. doi: 10.1371/journal.pone.0227612 (PMC7094877; doi:10.1371/journal.pone.0227612)
Supplement: S1 Protocol — (PDF) [file pone.0227612.s004.pdf]

STOPDAPT
ShorT and OPtimal duration of Dual AntiPlatelet Therapy study

<u>Protocol</u>

＜Date of preparation: Aug 20, 2012＞

Summary of the Study STOPDAPT

ShorT and OPtimal duration of Dual AntiPlatelet Therapy study after everolimus-eluting cobalt-chromium stent

[Exploratory study to evaluate the safety of reduction of thienopyridine treatment period to 3 months after implantation of Cobalt-Chromium everolimus-Eluting Stents (XIENCE Prime™)**]**

Summary of the Study: Patients who received PCI using drug-eluting stent XIENCE Prime™ are enrolled serially, if the doctor in charge judged that the period of postoperative thienopyridine therapy can be reduced to 3 months. Thienopyridine treatment is discontinued at 3 months after stent implantation, and incidences of cardiovascular events and bleeding events at 12 months after surgery are evaluated.
Since this study is aimed at evaluating the real clinical situation, no exclusion criteria are defined.
Patients who have received any drug-eluting stents other than cobalt-chromium everolimus-eluting stent(XIENCE/PROMUS™) in the past should be excluded.

- **Study design:** Multicenter prospective registry
- **Primary Endpoint:** Composite endpoint of cardiovascular death at 12 months, myocardial infarction (MI), stroke (ischemic and hemorrhagic), stent thrombosis (Definite stent thrombosis not resulting in MI) and serious bleeding (TIMI Major/Minor).
- **Primary endpoint evaluation:** Exploratory assessment is made after 3-month thienopyridine treatment on the achievement of the performance goal calculated based on the incidence of the primary endpoint at 12 months obtained from the everolimus-eluting stent (XIENCE™) group in the RESET study, in which 90% of the patients were treated with thienopyridine for at least 1 year.
- **Target sample size:** 1500 patients
- **Principal investigator:** Takeshi Kimura Department of Cardiovascular Medicine, Kyoto University
- **Clinical study managers:**

Kazushige Kadota	Kurashiki Central Hospital
Ken Kozuma	Teikyo University Hospital
Yoshihiro Morino	Iwate Medical University Hospital
Keiichi Igarashi	Hokkaido Social Insurance Hospital
Kengo Tanabe	Mitsui Memorial Hospital

Study Administration Staff:

Masahiro Natsuaki	Department of Cardiovascular Medicine, Kyoto University
Erika Yamamoto	Department of Cardiovascular Medicine, Kyoto University

- **Study period:** From August, 2012 to July, 2014 (planned)
- **Patient Enrollment Period:** From august, 2012 to July, 2013 (planned)

Index

LIST OF ABREVIATIONS (COMMON EXAMPLES) 5

1. STUDY OBJECTIVE 6

2. BACKGROUND AND RATIONALE 6

3. STUDY METHODS 7

4. RULES FOR STUDY IMPLEMENTATION 7

4.1 INCLUSION / EXCLUSION CRITERIA 7
4.2 ISSUED TO BE CONSIDERED 7
4.3 REQUIRED EXAMINATIONS 8
4.4 FOLLOW-UP PERIOD 8

5. STUDY PERIOD 9

6. ITEMS TO BE INVESTIGATED 9

6.1 PLANNED FOLLOW-UP PERIODS 9
6.2 OBSERVATION ITEMS 9
6.2.1 Observation Items at Enrollment 9
6.2.2 Follow-up at 12 months 11
6.2.3 Completion / Discontinuation of the Study 12

7. ENDPOINTS 13

7.1 PRIMARY ENDPOINT 13
7.2 SECONDARY ENDPOINTS 13
7.2.1 Major secondary endpoints 13
7.2.2 Secondary Endpoints 13

8. DETERMINATION OF SAMPLE SIZE 14

8.1 SAMPLE SIZE REQUIRED FOR SAFETY ASSESSMENT ON THE PRIMARY ENDPOINT 14

9. SUBGROUP ANALYSES 14

9.1 PRE-SPECIFIED SUBGROUP 14

10. OTHER NECESSARY ISSUES 15

10.1 ETHICAL CONCERNS/OBTAINMENT OF INFORMED CONSENT 15
10.1.1 Protection of patients' rights 16
10.1.2 Explanation to the patient 16
10.1.3 Privacy Issues 16
10.1.4 Compensation for health damages 16

10.1.5 Handling of treatment costs....17
10.2 PROTOCOL APPROVAL....17
10.3 PROTOCOL AMENDMENTS....17
10.4 DISCONTINUATION AND COMPLETION OF THE STUDY....17
10.5 DISCONTINUATION OF THE STUDY....17
10.6 TERMINATION OF THE STUDY....17
10.7 DEFINITIVE RATING OF ENDPOINTS....17
10.7.1 Clinical Endpoints - Clinical Events Committee (CEC)....17
10.7.2 Angiography Core Laboratory....18

11. DEFINITION OF ENDPOINTS....18

11.1 DEATH....18
11.2 MYOCARDIAL INFARCTION: MI....18
11.3 CORONARY REVASCULARIZATION....20
11.4 STENT THROMBOSIS....20
11.5 SURGERY....21
11.6 BLEEDING/HEMORRHAGIC COMPLICATIONS....22
11.7 COMPOSITE ENDPOINT....23
11.8 STROKE OR CEREBROVASCULAR ACCIDENT....24
11.9 CLASSIFICATION OF ANGINA....24

12. STUDY ORGANIZATION....24

12.1 PRINCIPAL INVESTGATOR....24
12.2 STUDY MANAGERS....25
12.3 STUDY COORDINATOR....25
12.4 STUDY ADMINISTRATION OFFICE....25
12.4 DATA CENTER....25
12.5 STATISTICAL ANALYSIS MANAGER....25
12.6 ANGIOGRAPHY CORE LABORATORY....26
12.7 SAFETY EVALUATION COMMITTEE MEMBERS....26
12.8 CLINICAL EVENTS COMMITTEE (CEC) MEMBERS....26
12.10 PARTICIPATING INSTITUTES....26
12.11 STUDY SPONSOR....26

14. AUTHORSHIP....26

15. REFERENCES....27

LIST OF ABREVIATIONS (COMMON EXAMPLES)

Abbreviation	Description
DES	Drug Eluting Stent
BMS	Bare-Metal Stent
DAPT	Dual AntiPlatelet Therapy
CABG	Coronary Artery Bypass Graft
PCI	Percutaneous Coronary Intervention
EES	Everolimus-Eluting Stent
LMCA	Left Main Coronary Artery
eGFR	estimated Glomerular Filtration Rate
ECG	Electrocardiogram
MI	Myocardial Infarction
ST	Stent Thrombosis
ACS	Acute Coronary Syndrome
QCA	Quantitative Coronary Angiography
TLR	Target Lesion Revascularization
TVF	Target Vessel Failure
TVR	Target Vessel Revascularization

1. STUDY OBJECTIVE

Safety of reduction of thienopyridine treatment period to 3 months after implantation of Cobalt-Chromium everolimus-eluting Stents (XIENCE Prime™) will be evaluated in an exploratory manner.

2. BACKGROUND AND RATIONALE

The first-generation drug-eluting stents (DES), sirolimus-eluting stent (sirolimus-eluting stent: SES) and Paclitaxel-eluting stent (paclitaxel -eluting stent: PES), have achieved a remarkable outcome with reduction of the restenosis rate at 1 year after coronary stent implantation to ≤10%, and DESs are currently used in the majority of percutaneous coronary interventions (PCI) (References 1 and 2). On the other hand, as a problem associated with the first-generation drug-eluting stents, incidence of late-onset adverse events, including very late stent thrombosis and late restenosis, occurring after 1 year postoperatively, which were rarely associated with conventional bare-metal stents (BMS) has been pointed out, although not frequently (References 3 and 4).

Thienopyridine antiplatelet agents (platelet P2Y12 receptor antagonists) have markedly inhibited incidence of stent thrombosis, when they were combined with aspirin for 1 month after implantation of BMS (References 5 and 6). On the other hand, combination of aspirin with thienopyridine (dual antiplatelet therapy: DAPT) for more than 1 year after BMS implantation is frequently used to prevent very late stent thrombosis in the current clinical practice. In the RESET (Randomized Evaluation of Sirolimus-eluting versus Everolimus-eluting stent Trial) study, which was carried out in clinical practice in Japan, DAPT was performed for at least 1 year in 90% of the patients (Reference 7). DAPT for 1-year is recommended in the labeling of XIENCE Prime™ that is an improvement from everolimus-eluting stent that is currently most widely used in Japan. However, there has been no report showing that long-term thienopyridine treatment for at least 1 year reduces incidence of serious cardiovascular events, such as late adverse event and death after DES implantation, myocardial infarction (MI) and stroke. In addition, severe bleeding complication such as cerebral hemorrhage by prolonged DAPT is detrimental to the patients. To clarify the optimal duration of DAPT after implantation of DES is very important issue for patients who received DES implantation. Large-scale observational studies or small-scale randomized comparative studies size have demonstrated that thienopyridine treatment for 6 months (References 8-10) or for at least 12 months (Reference 11) does not reduce incidence of serious cardiovascular events. To evaluate the efficacy of DAPT for 6 months or for at least 12 months, large-scale studies, such as ISAR-SAFE (The Intracoronary Stenting and Antithrombotic Regimen: Safety And Efficacy of 6 Months Dual Antiplatelet Therapy After Drug-Eluting Stenting) and DAPT (Dual Antiplatelet Therapy) are ongoing (References 12, 13). In addition, we recently reported that a 4-month landmark analysis in 6309 patients enrolled in the CREDO-Kyoto (Coronary Revascularization Demonstrating Outcome Study in Kyoto) PCI/CABG Registry Cohort-2 who had received DES (mainly SES) implantation revealed that death in the following 3 years and incidences of MI and stroke showed no difference between patients who had been continuing thienopyridine treatment and those who had discontinued it at 4 months, while incidence of hemorrhagic complications tended to be higher in patients who had been continuing thienopyridine treatment. These results suggest that the optimal duration of DAPT after DES implantation may be shorter than 6 months (Reference 14).

With respect to Everolimus-eluting stent (EES), it has been associated with significantly lower incidence of early or late (within 1-year) stent thrombosis compared with the first-generation DES such as PES and SES and with BMS in large-scale observational study (Reference 4) and randomized comparative studies (References 15-17) and their meta-analyses (References 18, 19). In addition to these reports, recently EES received CE Mark in Europe for a DAPT length of minimum 3 months based on the observational study of

EES implanted patients demonstrated that no cases of stent thrombosis at 1-year were reported in patients who discontinued DAPT after 3 months post-EES implantation.
Considering that long-term DAPT obviously increases hemorrhagic complications compared to Aspirin monotherapy (References 10, 20, 21), it is desirable to reduce the duration of DAPT as far as possible, if long-term DAPT is not effective in inhibiting the incidence of serious cardiovascular events. Moreover, long-term DAPT enormously increases medical expenses. In this study, we planned an exploratory multicenter study to evaluate incidences of cardiovascular events and bleeding events at 12 months after stent implantation using an EES (XIENCE Prime™), which is associated with low risk of stent thrombosis, when thienopyridine therapy is discontinued at 3 months after surgery.

3. STUDY METHODS

In this study, patients who received PCI using a drug eluting stent XIENCE Prime™ are enrolled serially, if the doctor in charge judged that the period of thienopyridine therapy can be reduced to 3 months. thienopyridine treatment is discontinued at 3 months after stent implantation, and incidences of cardiovascular events and bleeding events at 12 months after surgery are evaluated. Since this study is aimed at evaluating the real clinical situation, no exclusion criteria are defined. Patients who have received any drug-eluting stents other than everolimus-eluting cobalt-chromium stent（XIENCE /PROMUS™）in the past should be excluded. Exploratory assessment is made after 3-month thienopyridine treatment on the achievement of the performance goal calculated based on the incidence of the primary endpoint at 12 months obtained from the everolimus-eluting stent (XIENCE™) group in the RESET study, in which 90% of the patients were treated with thienopyridine for at least 1 year.

4. RULES FOR STUDY IMPLEMENTATION

4.1 Inclusion / Exclusion Criteria

- In this study, patients who received PCI using a drug eluting stent XIENCE Prime™ are enrolled serially, if the doctor in charge judged that the period of thienopyridine therapy can be reduced to 3 months.
- Patients who have received any drug-eluting stents other than everolimus-eluting cobalt-chromium stent（XIENCE /PROMUS™）in the past should be excluded.
- Patients who have received administrations of antiplatelet drugs other than aspirin / thienopyridine should be excluded.

4.2 Issued to be considered

- Enrollment should be carried out after PCI with XIENCE Prime™ during the same hospitalization.
- The type and dose of aspirin are chosen according to the institute’s guideline. The type and dose of thienopyridine should be equivalent to clopidogrel 75 mg/day. For patients who do not tolerate clopidogrel, this can be substituted with ticlopidine 200 mg/day.
- Administrations of antiplatelet drugs other than aspirin / thienopyridine are not acceptable.

- Patients can also be visited by the referring doctor, but Thienopyridine should be prescribed at the study institute.
- In discontinuing thienopyridine therapy, outpatient visit should be made in principle, and the discontinuation should be fully explained to the patient. F/U at 12 months after surgery should be performed at outpatient visit as far as possible. However, if the visit is impossible, F/U by telephone interview can be accepted. Incidence of any event should be recorded in the appropriate form.
- Although the planned follow-up period is 12 months, this can be prolonged if the Safety Evaluation Committee considers that safety must be followed up for a longer period.
- Acceptable time window for the discontinuation of thienopyridine therapy should be within ±1 month.
- To ensure adherence to the administration schedule of thienopyridine, CRC of the study institute or equivalent staff communicates to the outpatient doctor the necessity of discontinuing the prescription of thienopyridine at the outpatient visit at 3 to 4 months after PCI, and confirms that the prescription of thienopyridine has been discontinued at the same visit.
- While initial enrollment is done by the investigator of each institute, data input, including PCI form, should be carried out by the institute's CRC or external CRC.
- Each institute should report the patients' background (age, sex, diagnosis, presence of diabetes mellitus, renal function, and target lesion) and the planned period of DAPT treatment even for the patients who receive XIENCE Prime™ implantation in the enrollment period and have not received any drug-eluting stents other than everolimus-eluting cobalt-chromium stent（XIENCE /PROMUS™）in the past , and who are not enrolled in this study.
- If planned staged PCI is scheduled, the patient will be enrolled after completion of all PCI. The last PCI should be the index procedure.
- Qualitative angiographic analysis and QCA analysis before and after Index procedure will be performed in 300 patients randomly selected. QCA analysis should be performed by core laboratory.
- For follow-up coronary angiography, the guideline of each institute should be followed. When coronary angiography is planned, recurrence of angina symptoms and results of evaluation of ischemia should be recorded in the clinical record before angiography.
- Recurrence of angina symptoms and results of evaluation of ischemia at the time of TLR are recorded in the clinical record.

4.3 Required examinations

The following examinations are mandatory. Other examinations may be added according to each institute's standards.

At Enrollment:

- Blood tests
  - In this study, hemoglobin and hematocrit concentrations should be measured, because TIMI definition is used to rate hemorrhagic adverse events.
  - Hemoglobin and hematocrit concentrations should be measured also when a hemorrhagic adverse event is suspected during the follow-up period.
  - WBC, RBC, hemoglobin, hematocrit、Plt, creatinine, blood glucose、HbA1c, total cholesterol, HDL cholesterol, LDL cholesterol, triglyceride

4.4 Follow-up period

In this study, information described in “6. Items to be investigated” will be collected at enrollment and at 12 months and recorded on the electronic Case Report Form (eCRF).

5. STUDY PERIOD

This study will be carried out from August 2012 to July 2014, corresponding to the period from the beginning of enrollment to the scheduled completion of 12-month follow-up after PCI in the last enrolled patient. Although the enrollment period of this study is defined as within 1 year from August 1, 2012 to July 31, 2013, enrollment will be terminated when a total of 1200 patients are enrolled.

6. ITEMS TO BE INVESTIGATED

6.1 Planned Follow-up Periods

The investigations in this study are performed at the following two time points:

1) At enrollment
2) Follow-up at 12 months after procedure (At the outpatient visit in principle, but telephone interview is also accepted.)

Acceptable time windows for follow-up visits are not established. However, follow-up at 12 months is to be performed within ±1 month of the defined time point.

6.2 Observation Items

Observation items will be investigated at enrollment and at follow-up visit by various examinations and interview, etc. All the results will be recorded in the corresponding columns of the eCRF.

6.2.1 Observation Items at Enrollment

1. Enrollment data

Name of institute, date of enrollment, patient enrollment number, patient’s initials, and name of the investigator

2. Basic data

Age, sex, height, weight, date of hospitalization, blood pressure at hospitalization, pulse rate at hospitalization

3. Diagnosis of myocardial infarction (MI) or angina pectoris (select from below)

ST-elevation acute myocardial infarction (MI) , Non ST-elevation acute MI, stable angina pectoris, unstable angina pectoris, asymptomatic myocardial ischemia, old myocardial ischemia, coronary stenosis

4. History of cardiac diseases

History of PCI, history of CABG, history of myocardial infarction (MI), history of heart failure, history of stroke, history of atrial fibrillation, history of COPD, history of liver cirrhosis, history of malignant tumor, history of hemorrhagic disease

5. Complications

Complication of heart failure at hospitalization, heart failure (current or past history), carotid artery stenosis, disease of peripheral vessels excluding carotid arteries, aortic aneurism/dissection, diseases of the aorta/ peripheral vessels (all), diseases of the aorta/peripheral vessels (postoperative/planned to be treated), dialysis

6. Risk factors

Hypertension, Lipid abnormalities, smoking, diabetes mellitus, family history of coronary diseases

7. Concomitant medication

The presence or absence of anticoagulation therapy

8. Coronary angiographic findings

Number of branches affected; presence/absence of following treatments: treatment of unprotected left main coronary artery stenosis, treatment of chronic total occlusion, treatment of lesions responsible for ST-elevation acute MI, treatment of bifurcation lesion, treatment of 2 branches or more, treatment of 3 branches or more; left ventricular ejection fraction, measurement method for left ventricular ejection fraction, mitral insufficiency, evaluation method for mitral insufficiency.

9. Clinical laboratory tests

WBC、RBC、hemoglobin, hematocrit, Plt, creatinine, blood glucose, HbA1c, total cholesterol, HDL cholesterol, triglyceride

10. Cardiac enzymes

Creatine kinase (CK), creatine kinase-MB isoenzyme (CK-MB), troponin T, troponin I

11. Echocardiography (ECG)

Notes: Definition of observation items

1. Diabetes mellitus

Diabetes mellitus is defined as meeting either of 2 hour OGTT glucose level of ≥200 mg/dL, casual blood glucose level of ≥200 mg/dL, fasting blood glucose level of ≥126 mg/dL, or HbA1c ≥6.1% (JDS) or ≥6.5% (NGSP).

When the above tests have not been performer, patients who have been clinically diagnosed as diabetic or are treated with antidiabetic agents are defined as having diabetes.

2. Dyslipidaemia

Patients with total cholesterol ≥ 240 mg/dL or HDL cholesterol < 40 mg/dL, or patients who are treated with statins.

3. Evaluation of renal functions

The glomerular filtration rate (eGFR) is calculated by using the equation fitted for Japanese people by the Japanese Society of Nephrology.

eGFR=194*Cr $^{-1.094}$*Age $^{-0.287}$ (*0.739 for females)
Terminal renal failure: e-GFR < 30 mL/min/1.73 mm^2
Chronic kidney disease: e-GFR < 60 mL/min/1.73 mm^2

4. Other items
Other items will be considered based on the clinical diagnosis described on the clinical record.

6.2.2 Follow-up at 12 months

At 12 months after enrollment, the following data will be recorded.

1. Death
Investigation method to determine the patient's death/survival, date of the last confirmation of death/survival, presence/absence of death, date of death, classification of cause of death, cause of death

2. Other events than death
Investigation method for other events than death, date of the last confirmation of other events than death, presence/absence of other events than death

3. Myocardial infarction (MI)
Presence/absence of MI, date of onset, ST-elevation MI, Q-wave MI, relationship with stent thrombosis, ARC classification, presence/absence of evaluation of the maximum values of cardiac enzymes, date of measurement of cardiac enzymes, CK, CK-MB, troponin T, troponin I, lethality

4. ACS
Presence/absence of emergency hospitalization due to ACS, date of onset, ACS classification, relationship with stent thrombosis, lethality

5. Definite stent thrombosis according to ARC definition
Presence/absence of stent thrombosis, date of onset, situation of onset, presence/absence of evaluation of the maximum values of cardiac enzymes, date of testing, CK, CK-MB, troponin T, troponin I, presence/absence of Interim TVR trial, relationship with the surgical procedure, presence/absence of hemorrhagic complications before the onset of stent thrombosis, antiplatelet therapy (aspirin and thienopyridine drugs) at the onset of stent thrombosis, lethality

6. Probable stent thrombosis according to ARC definition
Presence/absence of stent thrombosis, date of onset, classification (unexplained death within 30days / MI in the target vessel area)

7. Possible stent thrombosis according to ARC definition
Presence/absence of stent thrombosis, date of onset

8. Stroke
Presence/absence of stroke, date of onset, classification of stroke, lethality

9. Heart failure
Presence/absence of hospitalization due to heart failure, date of onset

10. Ventricular fibrillation, persistent ventricular tachycardia
Presence/absence of hospitalization due to ventricular fibrillation or persistent ventricular tachycardia, date of onset

11. Hemorrhagic complications
Presence/absence of hemorrhagic complications, date of onset, site of hemorrhage, Nadir Hb, Nadir Ht, presence/absence of blood transfusion, presence/absence of drop in blood pressure, presence/absence of surgical hemostasis, TIMI classification, GUSTO classification, BARC classification

12. Surgery
Presence/absence of surgery, date of surgery, presence/absence of systemic anesthesia, surgical procedure, surgical area

13. CABG
Presence/absence of CABG, date of CABG

14. Other revascularizations than TLR
Presence/absence of other revascularizations than TLR, date of revascularization, method of revascularization, other TVRs than TLR, presence/absence of clinically indicated revascularization

15. TLR
Presence/absence of TLR, date of TLR, revascularization method, presence/absence of clinically indicated revascularization

16. Information about TLR lesion unit (to be recorded for each lesion)
Presence/absence of TLR, date of TLR, revascularization method, PCI device, presence/absence of clinically indicated revascularization, presence/absence of follow-up angiography, date of follow-up angiography, reason of follow-up angiography, evaluation method of follow-up angiography, presence/absence of restenosis in the main branch, presence/absence of reocclusion of the main branch, presence/absence of restenosis in collateral branches, presence/absence of reocclusion of collateral branches

17. Treatment discontinuation
Date of the last confirmation of thienopyridine treatment status, presence/absence of administration at the last confirmation date discontinuation of single drugs, date of discontinuation, reason of discontinuation, alternative drugs or measures taken, resumption of single drugs, date of resumption, discontinuation of thienopyridine treatments, date of discontinuation, reason of discontinuation, alternative drugs or measures taken, resumption of thienopyridine treatments, date of resumption, date of the last confirmation of aspirin treatment status, presence/absence of administration at the last confirmation date, discontinuation of aspirin treatment, date of discontinuation, reason of discontinuation, alternative drugs or measures taken, resumption of aspirin, date of resumption

6.2.3 Completion / Discontinuation of the Study

If the study was completed or prematurely discontinued, date of the last contact with the subject, if the subject had completed the study, reason of discontinuation are recorded in the corresponding columns of

eCRF. In addition, if the study was prematurely discontinued reason of premature discontinuation is recorded.

7. ENDPOINTS

7.1 Primary endpoint

The primary endpoint in this study is a composite endpoint of cardiovascular death in 12 months, myocardial infarction (MI), stroke (ischemic and hemorrhagic), stent thrombosis (Definite stent thrombosis [ST] not resulting in MI) and serious bleeding (TIMI Major/Minor).

7.2 Secondary endpoints

7.2.1 Major secondary endpoints

In this study, the following major secondary endpoints will be evaluated at 12 months after enrollment.

- Cardiovascular death/ MI/ stroke/ definite ST
- Major bleeding (TIMI Major/ Minor)

7.2.2 Secondary Endpoints

In this study, the following secondary endpoints will be evaluated at 12 months after enrollment.

- Death / MI
- Death
- Cardiovascular death/ MI
- Cardiovascular death
- MI
- Stroke
- ST (ARC definition)
- TLF
- TVF
- MACE
- Any TLR
- Clinically-driven TLR
- Non-TLR
- CABG
- Any TVR
- Any revascularization
- Bleeding complications

8. DETERMINATION OF SAMPLE SIZE

8.1 Sample size required for safety assessment on the primary endpoint

The primary endpoint in this clinical study is a composite endpoint of cardiovascular death, myocardial infarction (MI), stroke, stent thrombosis and serious bleeding at 12-month follow-up. The event rate for the primary endpoint in this single arm study will be compared against a Performance Goal (PG) of 6.6% using an exact test through the binominal distribution.

This PG of 6.6% is derived by adding a delta to the assumed true event rate. Based on the historical XIENCE V data in the RESET trial (4% with a sample size 1559; reference 7), the one-sided 80% confidence limit is approximately 4.4%. To account for variability associated with the observed rate of 4% in the RESET trial, the true rate in this proposed trial is assumed to be 4.4%. With a delta taken to be 2.2% (50% of the base rate of 4.4%), the final PG is 6.6% (4.4%+2.2%).

Given the following assumptions:
One-sided alpha 0.025
True rate 4.4% (event during hospitalization were excluded)
PG 6.6%
An exact test through binomial distribution is used to calculate the p-value
Power approximately 95%

An effective sample size of 1455 is required. Take into consideration of dropout cases, a total of approximately 1500 patients is needed.

In addition, for evaluating the primary endpoint, complementary analysis using Cox proportional hazard model also will be performed, to take into account the differences in patient background of the everolimus-eluting Stent (XIENCE™) group in RESET study.

9. SUBGROUP ANALYSES

In this study, patients with different backgrounds are expected to receive the treatment. For this reason, subgroup analyses for diabetes, multiple branch lesions, etc. will be performed, as well as the analysis including all the patients.

9.1 Pre-specified Subgroup

Patient units:

- Diabetes
- Insulin-treated diabetes
- Age (≧75 / < 75)
- Hemodialysis
- e-GFR < 30, Non-HD
- Oral anticoagulants

- History of hemorrhagic
- STEMI
- ACS
- Emergency procedure
- LMCA
- 2 vessel PCI
- 3 vessel PCI
- Total stent length category
- On-label/off-label

Lesion units:
- Bifurcation
- LMCA
- Multiple overlapping stent
- ISR of BMS and DES
- CTO
- STEMI
- ACS
- Emergency procedure
- Ostial RCA
- Small Vessel

Notes: definition of lesions

- Overlapping stent is dealt with as 1 lesion.
- When a stent is implanted in the left anterior descending coronary artery overlapped on another stent implanted for the left main coronary artery stenosis, these are considered to be two lesions in the left main coronary artery and in the left anterior descending coronary artery, respectively.
- Ostial lesion of the left anterior descending coronary artery that is not accompanied by significant stenosis in the left main coronary artery, but was stented from the left main trunk crossing over a circumflex branch, this is dealt with as one lesion at the ostium of the left anterior descending coronary artery instead of a left main trunk lesion.
- Bifurcation lesion is considered to be one lesion together with the side branch.
- Any lesion having a side branch of ≥ 2.2 mm in diameter by visual evaluation is defined as a bifurcation lesion.
- Any lesion localized within 3 mm from the ostium is defined as an ostial lesion.
- On-label lesion is defined as a lesion of ≤ 30 mm in length and 2.5-3.5 mm in lumen that has not been treated before. However, lesions responsible for a recent myocardial infarction, ostial lesions, bifurcation lesions, thrombotic lesions and highly calcified lesions are not defined as on-label lesions.
- Any lesion that does not meet the criteria for on-label lesion is defined as an off-label lesion.

10. OTHER NECESSARY ISSUES

10.1 Ethical concerns/Obtainment of informed consent

10.1.1 Protection of patients' rights

Compliance with the Declaration of Helsinki
Study investigators should carry out this study according to either of "the latest version of the Declaration of Helsinki" or "Ethical Guidelines for Clinical Studies (Public Notice of the Ministry of Health, Labor, and Welfare No. 255, amended on July 30, 2008)" that maximizes the protection of patients.

10.1.2 Explanation to the patient

Prior to enrollment, the investigator should give the patient the information document approved by the Ethics Committee (Attachment-9) with verbal explanation of details of the content. After the explanation, the consent form (Attachment-10) attached to the information document should be filled in with required data and be signed. The consent form completed with all required data should be duplicated in two copies. One copy will be kept by the patient, and another copy by the investigator. The original copy will be stored in the clinical chart.

10.1.3 Privacy Issues

The clinical record, test data, records regarding the patient's informed consent, etc. will be stored at each medical institute, and the Case Report Forms and other related documents will be stored by the Data Center of the Department of Cardiovascular Medicine, Kyoto University. These records will be disclosed when requested for audit, but the confidentiality will be protected. Moreover, these records should be stored so as to be retrieved when necessary.
All the staff involved in this study has the duty of confidentiality as data handlers and should have the maximum efforts to protect patients' personal information. When patients' personal information is provided to outside of the institute, the name of each patient will be converted in initials at each institute, so that the patient can be identified only by the responsible person of the institute. Therefore, the name of the subject will not be transmitted from the participating institute to the Central Administration Office and the Data Center. Moreover, while the number assigned to the patient on the clinical chart at each institute will be used as the Patient ID Number, this number will be automatically encrypted when entered on the web. Therefore, the patient's number on the clinical chart is not transmitted from the participating institute to the Central Administration Office and the Data Center.
For identification of the patient and inquiry to each institute, the encrypted patient ID number and the patient's initials will be used. With regard to the use of the patient's initials as an ID data in this study, the risk of leaking personal information through the patient's initials only is considered to be very low. If the patient's initials are not used, identification of the patient should totally depend on the personal information control system of the institute. For this reason, investigation itself probably cannot be implemented, because identification of the patient will be impossible.

10.1.4 Compensation for health damages

Compensation for health damages associated with this study will be done only when it is obligated by legal liability. Compensation for health damages, which is caused by the medical intervention itself and not associated with the clinical study, will be deliberated by each institute. Since the risk of health damages associated with this clinical study is expected to be very low, the Study Administration Office does not affiliate with any liability insurance.

10.1.5 Handling of treatment costs

All the examinations and treatments regarding this study will be basically within the range of daily clinical practice. Therefore, medical fees of the patients participating in the study are refunded by medical insurances. For some blood tests and imaging tests that are not covered by medical insurances will be refunded from the research funds.

10.2 Protocol Approval

This study will be carried out after examination and approval of the protocol by the Ethics Committee of each participating institute or equivalent organizations.

10.3 Protocol Amendments

If amendments of the protocol are required after implementation of the protocol, this should be communicated from the Central Administration Office to each institute interrupting the study. After the amended protocol is examined, the results of examination will be submitted to the Ethics Committee of each participating institute for its approval.

10.4 Discontinuation and Completion of the Study

In principle, the study will be continued until the target sample size is achieved at enrollment, and evaluation of all the patients completed. However, if adverse events clearly related with this study occurred, an independent Safety Evaluation Committee will discuss about the continuation of the study.

10.5 Discontinuation of the Study

If this study must be discontinued for a reason that occurred during the study, the principal investigator, after discussing with study managers, should promptly report the discontinuation of the study and its reason to the Ethics Committee of each institute by written form.

10.6 Termination of the Study

When enrollment of all the patients is completed, the principal investigator should notify the completion of enrollment to the investigator of each institute, and each institute terminates the enrollment. Moreover, when the completion of follow-up of all the patients is verified, the principal investigator should notify the completion of follow-up of the patients to the investigator of each institute. The investigator of each institute should submit the study completion report to the chief of the medical research group of affiliation.

10.7 Definitive Rating of Endpoints

10.7.1 Clinical Endpoints - Clinical Events Committee (CEC）

Clinical Events Committee (CEC) will carry out the definitive rating of all the clinical endpoints, and vascular and hemorrhagic adverse events.

10.7.2 Angiography Core Laboratory

Angiographic endpoints (pathological findings and qualitative analysis) will be rated by Angiography Core Laboratory.

11. Definition of Endpoints

11.1 Death

Death is defined based on the Academic Research Consortium (ARC) classification

- **Cardiac Death**
  Any death due to proximate cardiac cause (eg, myocardial infarction, low-output failure, fatal arrhythmia), unwitnessed death and death of unknown cause, and all procedure-related deaths, including those related to concomitant treatment, will be classified as cardiac death. In addition, any unexpected death even in patients with coexisting highly lethal disease (e.g., cancer) should be classified as cardiac, unless an unequivocal noncardiac cause can be established.
- **Vascular Death**
  Death caused by noncoronary vascular causes, such as cerebrovascular disease, pulmonary embolism, ruptured aortic aneurysm, dissecting aneurysm, or other vascular diseases.
- **Non-cardiovascular Death**
  Any death not meeting the criteria for cardiac death or vascular death, such as death caused by infection, malignancy, sepsis, pulmonary causes, accident, suicide, or trauma.

11.2 Myocardial Infarction: MI

Myocardial Infarction (MI) is defined by the ARC criteria. However, periprocedural MI will be evaluated by CKMB, because the evaluation by troponin is too sensitive.

- **Baseline MI evaluation**
  EEG showing ST elevation, development of new abnormal Q-wave, clinical symptoms specific to MI, troponin or CK-MB values exceeding the standard values
- **Periprocedural MI**
  - Occurrence of any of the following events within 48 hours after PCI procedure will be judged as MI.
    - CK-MB **≥ 3 times Upper Reference Limit (URL)** (CK-MB value exceeding URL before procedure is not considered as a new MI, but as MI at enrollment.)
    - Abnormal ECG (new Q-wave, left bundle branch block)
  - Occurrence of troponin **≥ 5 times URL** or CK-MB **≥ 5 times URL** within 72 hours after CABG procedure accompanied by any of the following criteria will be judged as

MI. (CK-MB value exceeding URL before procedure is not considered as a new MI, but as MI at enrollment.)

    - Abnormal ECG (new Q-wave, left bundle branch block)
    - New occlusion of coronary autografts or grafts
    - Reduction in living myocardium confirmed by diagnostic imaging

- **Spontaneous MI**
  - Occurrence of any of the following events at > 48 hours after PCI or > 72 hours after CABG will be judged as MI. Mi caused by revascularization procedures, such as TLR and TVR, is defined as periprocedural MI.
    - Abnormal ECG (new Q-wave, left bundle branch block)
    - Troponin or CK-MB value **> URL** (CK-MB value exceeding URL before procedure is not considered as a new MI, but as MI at enrollment.)
- **Sudden Death**
  - When death occurred before blood sampling for biomarker measurements or while biomarkers appeared to be increasing, MI will be judged according to the following criteria:
    - Clinical symptoms suggesting ischemia that are accompanied by one of the following:
      - New ST elevation or left bundle branch block
      - Thrombus determined by angiography or at autopsy
- **Reinfarction**
  - When after onset of MI stable or decreasing values are confirmed in 2 biomarker measurements, but 20% increase 3 to 6 hours is observed after the second measurement.
    - If biomarkers are increasing or have not yet reached the peak, data are insufficient to diagnose reinfarction.

Electrocardiographic Classification:

- **Classification based on Q-wave**
  - **Q-wave MI (QMI)**
    - Development of abnormal Q-waves confirmed in 2 or more contiguous leads with or without elevation in cardiac enzymes.
  - **Non-Q-wave MI (NQMI)**
    - All MIs not classified as Q-wave.
- **Classification based on ST segment。**
  - **ST-elevation myocardial infarction (MI)（STEMI）**
    - New or presumably new elevation of ST segment at J point in 2 or more contiguous leads. Cut-off point is ≥ 0.2 mV in V1, V2 and V3 leads and ≥ 0.1 mV in other leads.
  - **Non-ST elevation myocardial infarction (MI)（NSTEMI）**
    - MI that is not STEMI

11.3 Coronary Revascularization

Classification:

- **Target Lesion Revascularization (TLR)**
  PCI performed in the target lesion (within 5 mm of the stent edges), or CABG performed for restenosis of the target lesion or for treatment of other complications
- **Target Vessel Revascularization (TVR)**
  PCI performed in the target vessel or revascularization by CABG, including TLR
- **Target Vessel Revascularization-Remote (TVR-Remote)**
  Revascularization of a non-target lesion in the target vessel
- **Non Target Vessel Revascularization (Non-TVR)**
  Any revascularization in a vessel other than the target vessel
- **Non Target Lesion Revascularization (Non-TLR)**
  Any revascularization in a lesion other than the target lesion
  Non-TLR = TVR-Remote + Non-TVR

Clinically indicated coronary revascularization:

- The revascularization that meets the following criteria is considered as clinically indicated revascularization. Presence/absence of clinical findings is judged by the operator of the procedure before the revascularization.
  - Recurrence of angina pectoris, presumably related to the target vessel;
  - Objective signs of ischemia at rest or during exercise test (or equivalent), presumably related to the target vessel;
  - Signs of functional ischemia revealed by any invasive diagnostic test (e.g., Doppler flow velocity reserve [FVR], fractional flow reserve [FFR]);
  - Revascularization for ≥ 70% diameter stenosis even in the absence of the above-mentioned ischemic signs or symptoms.

11.4 Stent Thrombosis

Based on the ARC definition, Stent thrombosis is classified into definite, probable and possible according to the “probability”, and into acute, subacute late and very late according to timing of the onset.

- **Definite Stent Thrombosis**
  - Angiographic confirmation of stent thrombosis*:
    - The presence of a thrombus† that originates in the stent segment (including 5 mm of the stent edges) is revealed by angiography, and presence of at least one of the following criteria within a 48-hour time window is observed:
      - Acute onset of ischemic symptoms at rest
      - New ECG changes that suggest acute ischemia
      - Typical rise and fall in cardiac biomarkers (refer to definition of spontaneous MI)

        - Nonocclusive thrombus
            - Intracoronary thrombus is defined as a (spheric, ovoid, or irregular) noncalcified filling defect or lucency surrounded by contrast material (on 3 sides or within a coronary stenosis) seen in multiple projections, or persistence of contrast material within the lumen, or a visible embolization
        - Occlusive thrombus
            - TIMI 0 or TIMI 1 intrastent or proximal to a stent up to the most adjacent downstream side branch or main branch
    - Pathological confirmation of stent thrombosis:
        - Evidence of recent thrombus within the stent determined at autopsy or via examination of tissue retrieved following thrombectomy
- **Probable Stent Thrombosis**
    - When the following cases occurred after intracoronary stenting:
        - Any unexplained death within the first 30 days after procedure‡
        - Irrespective of the time after the index procedure, any MI in the territory of the implanted stent in the absence of any other obvious cause such as angiography or other lesions
- **Possible Stent Thrombosis**
    - Any unexplained death from 30 days after intracoronary stenting

* The incidental angiographic documentation of stent occlusion in the absence of clinical signs is not considered to be a confirmed stent thrombosis (silent occlusion)
† Intracoronary thrombus

- **Acute Stent Thrombosis**
  0-24 hours post stent implantation (Time 0 is defined as the time of removal of the guiding catheter).
- **Subacute Stent Thrombosis**
  > 24 hours-30 days post stent implantation
- **Late Stent Thrombosis ***
  > 30 days-1 year post stent implantation
- **Very Late Stent Thrombosis ***
  > 1 year post stent implantation

* Including “primary” as well as “secondary” stent thrombosis after stented segment revascularization.

11.5 Surgery

- Including endoscopic surgeries and therapies
- Including CABG

- Excluding percutaneous intravascular treatments
- Including aortic aneurysm stent graft procedure
- Excluding tooth extraction

11.6 Bleeding/Hemorrhagic Complications

Bleeding/Hemorrhagic Complications will be evaluated using the TIMI, GUSTO and BARC definitions (References 22-24).

TIMI bleeding classification:

Bleeding is classified by the Thrombosis in Myocardial Infarction (TIMI). Measurement of hemoglobin and hematocrit values at baseline is required for the severity rating.

- **Major Bleeding**
  - When any of the following criteria is met.
    - Intracranial hemorrhage
    - Decrease in hemoglobin to ≥ 5 g/dL decrease in the hemoglobin concentration
    - Absolute drop in hematocrit to ≥ 15% (Baseline – Onset of the event)
- **Minor Bleeding**
  - When blood loss is observed, and any of the following criteria is met:
    - Decrease in hemoglobin to ≥ 3 g/dL
    - Decrease in hematocrit to ≥ 10% (Baseline – Onset of the event)
  - When no blood loss is observed, but any of the following criteria is met:
    - Decrease in hemoglobin to ≥ 4 g/dL
    - Decrease in hematocrit to ≥ 12% (Baseline – Onset of the event)
- **Minimal Bleeding**
  - Any clinically overt sign of hemorrhage that is associated with a fall in hemoglobin to < 3 g/dL.
    (Microscopical urine occult blood and fecal occult blood are not defined as Minimal bleeding.)

GUSTO bleeding classification:

Severe Bleeding

- Life-threatening bleeding
- Intracranial hemorrhage
- Hemorrhage or bleeding that causes drop in blood pressure and requires interventions, such as infusion, blood transfusion, administration of a hypertensor, surgical interception.

Moderate Bleeding

- Bleeding that requires blood transfusion but does not meet criteria for severe bleeding

BARC bleeding classification:

Bleeding is classified based on definitions by the Bleeding Academic Research Consortium (BARC). Measurement of hemoglobin concentration is required for severity rating.

- **Type 0:** No bleeding

- **Type 1:** Bleeding that is not medically significant and does not cause the patient to seek unscheduled performance of studies, hospitalization, or treatment by a health care professional.
- **Type 2 :** Any overt sign of haemorrhage that should be treated and does not fit the criteria for Types 3, 4, or 5, but does meet at least one of the following criteria:
  (1) requiring non-surgical, medical intervention by a health care professional, (2) leading to hospitalization or increased level of care, (3) prompting evaluation.
- **Type 3 :**
  - Type 3a
    - Overt bleeding plus hemoglobin drop of 3-5 g/dL
    - Transfusion with overt bleeding
  - Type 3b
    - Overt bleeding plus hemoglobin drop of ≥ 5 g/dL
    - Cardiac tamponade
    - Bleeding requiring surgical intervention (excluding dental/nasal/skin/haemorrhoid)
    - Bleeding requiring intravenous vasoactive drugs
  - Type 3c
    - Intracranial hemorrhage
    - Intraocular bleeding compromising vision
- **Type4:** CABG-related bleeding
  - Perioperative intracranial hemorrhage within 48 hours
  - Reoperation following closure of sternotomy for the purpose of controlling bleeding
  - Transfusion of ≥ 5 units of whole blood or concentrated red blood cell within 48 hours
  - Chest tube output ≥ 2 L within 48 hours
- **Type5:** Fatal bleeding
  - Type 5a
    Probable Fatal bleeding: no autopsy or imaging confirmation, but clinically suspicious
  - Type 5b
    Definite fatal bleeding: overt bleeding or autopsy or imaging confirmation

11.7 Composite endpoint

The following categories are defined as composite endpoint:

- **Target Lesion Failure (TLF)**
  Cardiac death, myocardial infarction (MI) of target vessels, Clinically indicated target lesion revascularization
- **Tagert Vessel Failure (TVF)**
  Cardiac death, myocardial infarction (MI), Clinically indicated target vessel revascularization
- **Major Adverse Cardiac Events (MACE)**
  Cardiac death, myocardial infarction (MI), Clinically indicated target lesion revascularization

11.8 Stroke or Cerebrovascular Accident

Acute onset of a neurological deficit that persists for at least 24 hours and is the result of a disturbance of the cerebral circulation due to ischemia or hemorrhage.

Deficits that last ≤ 24 hours are due to transient ischemic neurological attack and are not classified in this category.

11.9 Classification of Angina

- **Braunwald Classification of Unstable Angina (Reference 25)**
  - **Class I**: New onset of severe or accelerated angina: Patients with new onset (< 2 months in duration) exertional angina pectoris that is severe or frequent (> 3 episodes/day) or patients with chronic stable angina who develop accelerated angina (angina distinctly more frequent, severe, longer in duration, or precipitated by distinctly less exertion than previously) but who have not experienced pain at rest during the preceding 2 months.
  - **Class II**: Angina at rest, subacute: Patients with 1 or more episodes of angina at rest during the preceding month but not within the preceding 48 hours
  - **Class III**: Angina at rest, acute: Patients with 1 or more episodes of angina at rest within the preceding 48 hours

Canadian Cardiovascular Society (CCS) Classification of Stable Angina (Reference 26)

- **Class I**: Ordinary physical activity does not cause angina, such as walking or climbing stairs. Angina occurs with strenuous, rapid or prolonged exertion at work or recreation.
- **Class II**: Slight limitation of ordinary activity. Angina occurs on walking or climbing stairs rapidly, walking uphill, walking or stair climbing after meals, or in cold, in wind, under emotional stress or only during the few hours after awakening. Angina occurs on walking more than two blocks on the level and climbing more than one flight of ordinary stairs at a normal pace and in normal condition.
- **Class III**: Marked limitation of ordinary physical activity. Angina occurs on walking one to two blocks on the level and climbing one flight of stairs in normal conditions and at a normal pace.
- **Class IV**: Inability to carry on any physical activity without discomfort – angina symptoms may be present at rest.

12. STUDY ORGANIZATION

12.1 Principal investgator

Takeshi Kimura Department of Cardiovascular Medicine, Kyoto University

12.2 Study Managers

Kazushige Kadota	Kurashiki Central Hospital
Ken Kozuma	Teikyo University Hospital
Yoshihiro Morino	Iwate Medical University Hospital
Keiichi Igarashi	Hokkaido Social Insurance Hospital
Kengo Tanabe	Mitsui Memorial Hospital

Study Administration Staff

Masahiro Natsuaki	Department of Cardiovascular Medicine, Kyoto University
Erika Yamamoto	Department of Cardiovascular Medicine, Kyoto University

12.3 Study Coordinator

Junya Ako	Saitama Medical Center Jichi Medical University
Takashi Ueno	Kurume University Hospital
Yoshihisa Nakagawa	Tenri Yorozu Hospital
Junji Yajima	The Cardiovascular Institute Hospital
(Offering)	

12.4 Study Administration Office

Research Institute for Production Development
15 Morimoto-cho, Shimogamo, Sakyo-ku, Kyoto 606-0805, Japan
Tel: 075-781-1107 Fax：075-791-7659
Person in charge of study administration: Naoko Okamoto
Cardiovascular Research Promotion Unit
Person in charge of contracts: Kumiko Kitagawa
General Affairs Department

12.4 Data Center

Department of Cardiovascular Medicine, Kyoto University Graduate School of Medicine
54 Shogoin Kawahara-cho, Sakyo-ku, Kyoto 606-8507, Japan
Tel: 075-751-4255 Fax：075-751-3299
Responsible person: Takeshi Kimura

12.5 Statistical Analysis Manager

Takeshi Morimoto	Center for Medical Education, Kyoto University

12.6 Angiography Core Laboratory

Cardio Core Japan

201 Sky Plaza, 4-20-8, Kamijujo, Kita-ku, Tokyo 114-0034

Tel: 03-5993-9140 Fax：03-5993-9140

Person in charge: Ken Kozuma

12.7 Safety Evaluation Committee Members

Tadanori Aizawa	The Cardiovascular Institute Hospital
Tetsu Yamaguchi (Offering)	Toranomon Hospital

12.8 Clinical Events Committee (CEC) members

Yutaka Furukawa	Kobe City Medical Center General Hospital
Mitsuru Abe (Offering)	National Hospital Organization Kyoto Medical Center

12.10 Participating institutes

To be determined

12.11 Study Sponsor

Abbott Vascular Japan, Co., Ltd.

The study sponsor participated in the discussion for preparation of the study protocol, but is not involved in the implementation of the study, data collection, event fixation and statistical analysis. However, approval of the study sponsor should be obtained for presentation in scientific meetings and submission of papers. The study sponsor has a non-exclusive right to use all the information or data obtained in this study.

14. AUTHORSHIP

Main paper： Takeshi Kimura

For other sub-analyses than those described above, topics proposed from the institutes are selected in order of the number of enrolled patients.

15. REFERENCES

1) Moses JW, Leon MB, Popma JJ, et al. Sirolimus-eluting stents versus standard stents in patients with stenosis in a native coronary artery. N Engl J Med. 2003;349:1315-23.

2) Stone GW, Ellis SG, Cox DA, et al. A polymer-based, paclitaxel-eluting stent in patients with coronary artery disease. N Engl J Med. 2004;350:221-31.

3) Kimura T, Morimoto T, Nakagawa Y, et al. Very late stent thrombosis and late target lesion revascularization after sirolimus-eluting stent implantation: five-year outcome of the j-Cypher Registry. Circulation. 2012;125:584-91.

4) Raber L, Magro M, Stefanini GG, et al. Very late coronary stent thrombosis of a newer-generation everolimus-eluting stent compared with early-generation drug-eluting stents: a prospective cohort study. Circulation. 2012;125:1110-112.

5) Schoming A, Neumann FJ, Kastrati A, et al. A randomized comparison of antiplatelet and anticoagulant therapy after the placement of coronary-artery stents. N Engl J Med. 1996;334:1084-89.

6) Leon MB, Baim DS, Popma JJ, et al. A clinical trial comparing three antithrombotic-drug regimens after coronary-artery stenting. Stent Anticoagulation Restenosis Study Investigators. N Engl J Med. 1998;339:1665-71.

7) Kimura T, Morimoto T, Natsuaki M, et al. Comparison of Everolimus-eluting and Sirolimus-eluting Coronary Stents: 1-year outcomes from the Randomized Evaluation of Sirolimus-eluting versus Everolimus-eluting stent Trial (RESET). Circulation, in press.

8) Kimura T, Morimoto T, Nakagawa Y, et al. Antiplatelet therapy and stent thrombosis after sirolimus-eluting stent implantation. Circulation. 2009;119:987-95.

9) Gwon HC, Hahn JY, Park KW, et al. Six-month versus 12-month dual antiplatelet therapy after implantation of drug-eluting stents: the Efficacy of Xience/Promus Versus Cyper to Reduce Late Loss After Stenting (EXCELLENT) randomized, multicenter study. Circulation. 2012;125:505-13.

10) Valgimigli M, Campo G, Monti M, et al. Short- Versus Long-term Duration of Dual Antiplatelet Therapy After Coronary Stenting: A Randomized Multicentre Trial. Circulation. 2012 Mar 21. [Epub ahead of print]

11) Park SJ, Park DW, Kim YH, et al. Duration of dual antiplatelet therapy after implantation of drug-eluting stents. N Engl J Med. 2010;362:1374-82.

12) Byrne RA, Schulz S, Mehilli J, et al. Rationale and design of a randomized, double-blind, placebo-controlled trial of 6 versus 12 months clopidogrel therapy after implantation of a drug-eluting stent: The Intracoronary Stenting and Antithrombotic Regimen: Safety And Efficacy of 6 Months Dual Antiplatelet Therapy After Drug-Eluting Stenting (ISAR-SAFE) study. Am Heart J. 2009;157:620-4.

13) Mauri L, Kereiakes DJ, Normand SL, et al. Rationale and design of the dual antiplatelet therapy study, a prospective, multicenter, randomized, double-blind trial to assess the effectiveness and safety of 12 versus 30 months of dual antiplatelet therapy in subjects undergoing percutaneous coronary intervention with either drug-eluting stent or bare metal stent placement for the treatment of coronary artery lesions. Am Heart J. 2010;160:1035-41.

14) Tada T, Natsuaki M, Morimoto T, et al. Duration of Dual Antiplatelet Therapy and Long-term Clinical Outcome after Coronary Drug-eluting Stent Implantation: landmark analyses from the CREDO-Kyoto PCI/CABG Registry Cohort-2. Circ Cardiovasc Interv, in press.

15) Stone GW, Rizvi A, Newman W, et al. Everolimus-eluting versus paclitaxel-eluting stents in coronary artery disease. N Engl J Med. 2010;362:1663-74.

16) Kedhi E, Joesoef KS, McFadden E, et al. Second-generation everolimus-eluting and paclitaxel-eluting stents in real-life practice (COMPARE): a randomised trial. Lancet. 2010;375:201-9.

17) Sabate M. A clinical evaluation of Xience-V stent in acute myocardial infarction: the EXAMINATION trial. Presentation at Congress of European Society of Cardiology; Aug 27-31,2011; Paris, France.

18) Baber U, Mehran R, Shama SK, et al. Impact of the everolimus-eluting stent on stent thrombosis: a meta-analysis of 13 randomized trials. J Am Coll Cardiol. 2011;58:1569-77.

19) Palmerini T, Biondi-Zoccai G, Riva DD, et al. Stent thrombosis with drug-eluting and bare-metal stents: evidence from a comprehensive network meta-analysis. Lancet. 2012, Mar 22. [Epub ahead of print]

20) Berger PB, Bhatt DL, Fuster V, et al. Bleeding complications with dual antiplatelet therapy among patients with stable vascular disease or risk factors for vascular disease: results from the Clopidogrel for High Atherothrombotic Risk and Ischemic Stabilization, Management, and Avoidance (CHARISMA) trial. Circulation. 2010;121:2575-83.

21) Toyoda K, Yasaka M, Iwade K, et al. Dual antithrombotic therapy increases severe bleeding events in patients with stroke and cardiovascular disease: a prospective, multicenter, observational study. Stroke. 2008;39:1740-5.

22) Rao AK, Pratt C, Berke A, et al. Thrombolysis in Myocardial Infarction (TIMI) trial: phase I: hemorrhagic manifestations and changes in plasma fibrinogen and the fibrinolytic system in patients treated with recombinant tissue plasminogen activator and streptokinase. J Am Coll Cardiol. 1988;11:1-17.

23) The GUSTO investigators. An international randomized trial comparing four thrombolytic strategies for acute myocardial infarction. N Engl J Med 1993;329:673-82.

24) Mehran R, Rao SV, Bhatt DL et al. Standardized bleeding definitions for cardiovascular clinical trials: a consensus report from the Bleeding Academic Research Consortium. Circulation. 2011;123:2736-47.

25) Braunwald E. Unstable angina: a classification. Circulation 1989;80:410-14.

26) Campeau L. Letter: grading of angina pectoris. Circulation. 1976;54:522-3.
