# Supplementary material for: One-year clinical outcomes of patients with versus without acute coronary syndrome with 3-month duration of dual antiplatelet therapy after everolimus-eluting stent implantation
Source: PLoS One. 2020 Mar 25;15(3):e0227612. doi: 10.1371/journal.pone.0227612 (PMC7094877; doi:10.1371/journal.pone.0227612)
Supplement: S1 Appendix — (DOCX) [file pone.0227612.s005.docx]

**Lists.**

**List A. List of the participating centers and the investigators**

Saiseikai Kumamoto Hospital: Koichi Nakao, Shinzo Miyamoto

Kyoto University Hospital: Takeshi Kimura, Masahiro Natsuaki, Erika Yamamoto

Saitama Cardiovascular and Respiratory Center: Tetsuya Ishikawa, Joshi Tsutsumi

Chikamori Hospital: Kazuya Kawai, Shuichi Seki

Osaka City General Hospital: Kei Yunoki, Akira Itoh

Mashiko Hospital: Shogo Shimizu

National Hospital Organization Kyoto Medical Center: Masaharu Akao, Mitsuru Ishii

Mitsubishi Kyoto Hospital: Shinji Miki, Tetsu Mizoguchi, Masashi Kato

Kimitsu Chuo Hospital: Masashi Yamamoto

Seirei Hamamatsu General Hospital: Hisayuki Okada

Nagai Hospital: Kozo Hoshino

Teine Keijinkai Hospital: Mitsugu Hirokami,

Juntendo University Shizuoka Hospital: Satoru Suwa

Saiseikai Yokohamashi Tobu Hospital: Toshiya Muramatsu, Norihiro Kobayashi

Okamura Memorial Hospital: Yasuhiro Tarutani

Osaka Red Cross Hospital: Tsukasa Inada, Fujio Hayashi

Iwate Medical University Hospital: Yoshihiro Morino, Yu Ishikawa

Mie University Hospital: Masaaki Ito, Takashi Tanigawa, Toshiki Sawai

Kurashiki Central Hospital: Kazushige Kadota, Hiroyuki Tanaka

Shiga Medical Center for Adults: Shigeru Ikeguchi, Masaharu Okada, Yasutaka Inuzuka

Saiseikai Fukuoka General Hospital: Takeshi Serikawa, Toshiyuki Kozai, Masahiro Natsuaki

Mitsui Memorial Hospital: Kengo Tanabe, Takuya Hashimoto

Caress Sapporo Tokeidai Memorial Hospital: Kazushi Urasawa, Ryoji Koshida

Cardiovascular Center Hokkaido Ohno Hospital: Takehiro Yamashita, Taishi Maeno

Yokohama City University Medical Center: Kazuo Kimura, Kiyoshi Hibi

Kyoto Second Red Cross Hospital: Hiroshi Fujita, Koji Isodono

Hirakata Kohsai Hospital: Shoji Kitaguchi, Yuko Morikami

Hamamatsu Medical Center: Masakazu Kobayashi, Terumori Sato

Kobe City Medical Center General Hospital: Makoto Kinoshita

Japan Community Health Care Organization Hokkaido Hospital: Keiichi Igarashi, Jungo Furuya

Tenri Hospital: Yoshihisa Nakagawa, Toshihiro Tamura

Tokushima Red Cross Hospital: Koichi Kishi

Sakakibara Memorial Hospital: Tetsuya Tobaru, Itaru Takamisawa

Hyogo Prefectural Amagasaki Hospital: Yoshiki Takatsu, Ryoji Taniguchi

Hoshi General Hospital: Yoshitane Seino, Yasuhiro Shimizu

Kokura Memorial Hospital: Kenji Ando, Kyohei Yamaji

Takeda Hospital: Noriyuki Kinoshita

The Cardiovascular Institute Hospital: Junji Yajima, Nobuhiro Murata

Yokohama City University Hospital: Teruyasu Sugano, Hideyuki Ogawa, Masayoshi Kiyokuni

Japanese Red Cross Society Wakayama Medical Center: Takashi Tamura, Kousuke Takahashi

University of Occupational and Environmental Health Japan: Shinjo Sonoda, Kuninobu Kashiyama

Teikyo University Hospital: Hiroyuki Kyono

Fukuyama Cardiovascular Hospital: Hideo Takebayashi, Yuetsu Kikuta

National Cerebral and Cardiovascular Center Hospital: Satoshi Yasuda, Hiroki Sakamoto, Yasuhide Asaumi

Osaka City University Hospital: Minoru Yoshiyama, Takao Hasegawa, Tomokazu Iguchi

Wakayama Medical University Hospital: Takashi Akasaka, Tomoyuki Yamaguchi

Juntendo University Hospital: Katsumi Miyauchi, Shinya Okazaki

Matsue Red Cross Hospital: Kinya Shirota

Bell Land General Hospital: Toru Kataoka, Yuya Sakamoto

Kinki University Hospital: Shunichi Miyazaki, Masakazu Yasuda

Sendai Open Hospital: Atsushi Kato, Kenya Saji

Toyohashi Heart Center: Takahiko Suzuki, Yoshihisa Kinoshita

Aichi Medical University Hospital: Tetsuya Amano, Hiroaki Takashima

Saiseikai Matsuyama Hospital: Kouki Watanabe, Susumu Shigemi

Mie Heart Center: Hideo Nishikawa, Hiroyuki Suzuki

Tokyo Women's Medical University Hospital: Junichi Yamaguchi, Kazuho Kamishima

Saitama Medical Center Jichi Medical University: Junya Ako, Takuji Katayama, Wada Hiroshi

Sumitomo Hospital: Hisatoyo Hiraoka, Yuji Yasuga

**List B. Study Organization.**

**Steering Committee:**

Takeshi Kimura (Principal Investigator), Kazushige Kadota, Ken Kozuma, Yoshihiro Morino, Keiichi Igarashi and Kengo Tanabe.

**Advisory Committee:**

Junya Ako, Takashi Ueno, Yoshihisa Nakagawa and Junji Yajima.

**Clinical Event Committee:**

Yutaka Furukawa and Mitsuru Abe.

**Statistical Analysis:**

Takeshi Morimoto.

**Data safety monitoring board:**

Tadanori Aizawa and Tetsu Yamaguchi.

**Coordinating Center:** Research Institute for Production Development, Kyoto, Japan
Naoko Okamoto, Miya Hanazawa, Kumiko Kitagawa, Makoto Ishikawa, Misato Yamauchi, Yumika Fujino, Saori Tezuka, Yuki Sato, Chikako Hibi, Hitomi Sasae, Emi Takinami, Yuriko Uchida, Yuko Yamamoto, Satoko Nishida, Asuka Takahashi, and Yui Kinoshita.

**Clinical research coordinator:**

Iwate Medical University Hospital: Yumiko Okuyama

Sakakibara Memorial Hospital: Nobuko Okamura, Hiromi Shinoki

Mitsui Memorial Hospital: Rika Yamamoto

Tokyo Women's Medical University Hospital: Miyoko Naganuma

Hamamatsu Medical Center: Mariko Hirai

National Cerebral and Cardiovascular Center Hospital: Mihoko Uotani

Tenri Hospital: Miho Nakagawa

Wakayama Medical University Hospital: Natsuko Yumine

Kurashiki Central Hospital: Noriko Makita
