# Supplementary material for: One-year clinical outcomes of patients with versus without acute coronary syndrome with 3-month duration of dual antiplatelet therapy after everolimus-eluting stent implantation
Source: PLoS One. 2020 Mar 25;15(3):e0227612. doi: 10.1371/journal.pone.0227612 (PMC7094877; doi:10.1371/journal.pone.0227612)
Supplement: Attachment — Submitted filename: Responses to reviewers STOPDAPT ACS final.docx [file pone.0227612.s006.docx]

**Responses to the editor’s and reviewers’ comments**

We appreciate the kind editing and comments from the editor and the reviewers.

We amended the manuscript as suggested by the editor and reviewers.

**Editor:**

**I have concerns about the real need to compare two different populations from two different trials. Probably this part of the study should be omitted, else a complex propensity matching between patients from the different trials should be performed.**

We appreciate the comments.

As the editor suggested, we omitted the comparison of two different populations from the STOPDAPT and RESET trials.

**Reviewer #1:**

The submitted manuscript is a post-hoc analysis of the STOPDAPT trial, investigating the safety of a 3-month DAPT regimen after successful CoCr-EES implantation in patients with or without ACS. The authors aimed at evaluating if a short DAPT regimen is similarly safe in SCAD and ACS patients since those with an acute presentation have been traditionally considered at higher risk for recurrent thrombotic events and so in need for a more prolonged (at least 1-year) DAPT. The trial has a valid scientific ground, and methods and analyses are rigorously conducted. The authors concluded that “Stopping DAPT at 3-month after CoCr-EES implantation in patients with ACS was as safe as that in patients with stable CAD.”

**Detailed comments to be considered:

- In the main paper published in 2016, a sub-group analysis investigating the ACS vs. non-ACS group already showed the absence of a significant interaction between clinical presentation and outcomes. Thus, these results are only confirmatory of previously published data. However, taking into account the recognized limits of sub-group analyses, the presented data are of potential interest addressing this issue with a more rigorous and detailed approach.**

We appreciate the comments.

As reviewer commented, a sub-group analysis investigating the ACS vs. non-ACS group already showed the absence of a significant interaction between clinical presentation and outcomes. However, sub-group analyses are limited, and detail information regarding the baseline characteristics or clinical outcomes between the 2 groups would be available in this study.

**- The rate of the primary endpoint events was similar in the two cohorts of patients. However, while the rate of events in the stable CAD group (3%) is in line with recent epidemiological studies, the rate of 1-year events for the ACS (2.3%) appears unusually low as compared with other reports, and less than half of the RESET cohort (4.8%) that was used as historical reference by the authors. Surprisingly, the rate of events results even higher (although only numerically) in the SCAD group compared with the ACS, which is difficult to justify biologically. This difference is even more evident at the land-mark analysis, where the risk of future events in SCAD is almost twice of that in ACS (2.4% s. 1.3%). These results might be at least in part related to the high prevalence of patients with unstable angina in the ACS group. Indeed, 1,266 patients out of 1,525 (83% of the total population) had a non-MI presentation, with only 44 patients having an NSTEMI. For this reason, no conclusions should be drawn for the ACS “troponin-positive” patients, which actually represent those at higher risk of recurrent thrombotic events and for which more concerns exist in the case of premature DAPT discontinuation. Accordingly, the present results cannot be applied to the whole ACS spectrum but limited to those at low-risk of event recurrence (comparable to those with SCAD). The authors should carefully discuss this point in the manuscript and be more cautious with their conclusions.**

We appreciate the comments.

As reviewer commented, the rate of 1-year events for the ACS (2.3%) appears unusually low as compared with other reports, and less than half of the RESET cohort (4.8%) that was used as historical reference. These results might be at least in part related to the high prevalence of patients with unstable angina in the ACS group. Furthermore, the STOPDAPT trial had mostly enrolled stable patients after PCI, which might be one of the reasons for the low event rate even in ACS patients. Therefore, we added the following statement in the discussion section.

“In the present post-hoc analysis of the STOPDAPT trial demonstrated that 3-month DAPT after CoCr-EES implantation was associated with similarly low event rate for the primary endpoint without any definite ST events in both ACS and stable CAD patients, although 47% of ACS patients had troponin-negative UA at low-risk of event recurrence. Furthermore, the STOPDAPT trial had mostly enrolled stable patients after PCI, which might be one of the reasons for the low event rate even in ACS patients.” (Page 14, Line 250)

Furthermore, we amended the conclusion as follows.

“Stopping DAPT at 3-month after CoCr-EES implantation in patients with ACS including 47% of unstable angina was as safe as that in patients with stable CAD.”(Page 3, Line 49, Page 15, Line 275)

**- It is not clear which proportion of patients received intracoronary imaging to guide PCI. In the STOPDAPT-2, the (same) investigators reported a high rate of intracoronary imaging guidance according to the clinical practice in Japan. They stated "...the vast majority of patents in this study underwent PCI guided by intracoronary imaging devices, which are rarely used in the United States and Europe. Therefore, caution is warranted in extrapolating the current study results outside of Japan". Please, clarify the percentage of patients receiving intracoronary imaging in the present analysis, also reporting data for stable CAD and ACS patients separately (if available).**

We appreciate the comments.

As reviewer commented, the vast majority of patients underwent PCI guided by intracoronary imaging devices in the STOPDAPT-2. Therefore, it is important to clarify which proportion of patients received intracoronary imaging in this study. However, we do not have information on the proportion of patients who received intracoronary imaging in the STOPDAPT-1.

**- Introduction: “However, it is unclear whether the distinction between ACS and stable CAD regarding the recommendation on the DAPT duration was based on a solid scientific rationale.” This sentence should be probably reworded, as the scientific rationale for recommending a longer DAPT in patients with ACS, compared with those with stable CAD, is robust and based on the higher risk of future thrombotic events.**

We appreciate the comments.

As reviewer commented, longer DAPT in patients with ACS, compared with those with stable CAD, is based on the higher risk of future thrombotic events. Therefore, we deleted the following statement.

“it is unclear whether the distinction between ACS and stable CAD regarding the recommendation on the DAPT duration was based on a solid scientific rationale.”
 **- “Ethics” section: The full list of the individual centers participating in the trial can be reported in the supplementary appendix.**

We reported the full list of the individual centers participating in the trial in the supplementary appendix.

**- “Procedures” section: “Antiplatelet regimen included aspirin (≥81mg daily) …”. Which was the maximum dose of daily aspirin allowed in the trial? Did any patients receive ASA >100 mg? Please, report these data (if available).**

We appreciate the comments.

Only 13 patients (0.98%) had aspirin 200mg as the antiplatelet regimen. Therefore, vast majority of patients had aspirin 81-100mg daily.

**- All patients enrolled in the trial were treated with clopidogrel. Please, briefly discuss the absence of patients receiving ticagrelor/prasugrel.**

We appreciate the comments.

Ticagrelor/prasugrel were not available in the enrollment period between September 2012 and October 2013 in Japan. We added the following statement in the limitation section.

“Third, patients received clopidogrel as a P2Y12 receptor blocker even in patients with ACS. Prasugrel or ticagrelor were not available in the enrollment period between 2012 and 2013 in Japan. ” (Page 15, Line 268)

**- Endpoint and definition: In the era of high-sensitivity troponin, the diagnosis of UA is becoming rarer than in the past. Please clarify in the text which cardiac biomarkers were used for distinguishing UA vs NSTEMI, and how troponin was evaluated in the study (high-sensitivity?). Were the same methods for dosing cardiac biomarkers used across the 58 centers involved in the study (or not)? This might be a potential source of confounding in a multicentre study.**

We appreciate the comments.

We used high-sensitivity troponin or CK-MB for the diagnosis of MI. High-sensitivity troponin or CK-MB value > upper reference limit were diagnosed as MI in the 58 centers. We added the following statement in the method section.

“High-sensitivity troponin or CK-MB value > upper reference limit were diagnosed as MI in the all participating centers.” (Page 6, Line 108)

**-Moreover, the proportion of UA patients appears very high in what the authors define as an all-comers population, also if compared with the STOPDAPT-2 population. The authors report in the flow-chart of the study that 2,054 patients were excluded (actually, more than those enrolled). Can the authors clarify which was the proportion of patients with an ACS (UA, STEMI, and NSTEMI) or SCAD in the "non-enrolled" population? We can assume most of these patients were excluded because presenting with STEMI or NSTEMI and then considered by the physicians at high-risk of complications if treated with a short DAPT. This (prudent) attitude by the investigators might have biased patients enrollment, resulting in a "low-risk" study population (substantially including UA and SCAD, and excluding STEMI and NSTEMI), for which the conclusions should be applied. In this context, it would be of interest if the authors could provide the GRACE score of the NSTE-ACS patients.**

We appreciate the comments.

As reviewer suggested, we compared the proportion of patients with an ACS (UA, STEMI, and NSTEMI) or SCAD between enrolled and non-enrolled population. Patients with ACS, STEMI and NSTEMI patients were more included in the non-enrolled group than in the enrolled group. We include following statement in the results and limitation section.

“Baseline characteristics were significantly different in several aspects between the enrolled and non-enrolled patients (S1 Table 1). AMI presentation were more prevalent in the non-enrolled group, while stable CAD were more often found in the enrolled group.” (Page 9, Line 158)

“First, selection bias toward inclusion of patients with lower ischemic risk should be considered when interpreting the result of this study. Despite the all-comer study design, those ACS patients with large thrombotic burden and/or extensive atherosclerotic burden might well have not been included in this short DAPT trial. Indeed, patients with STEMI and NSTEMI were more often included in the non-enrolled group.” (Page 14, Line 262)

**S1 Table. Patient Characteristics: Enrolled versus Non-enrolled Patients**

|  | Enrolled | Non-enrolled | P Value |
| --- | --- | --- | --- |
|  | N=1525 | N=2054 |  |
| Age –years | 70.0±10.6 | 70.0±11.0 | 0.97 |
| Age >=75 years | 570 (37%) | 776 (38%) | 0.81 |
| Male gender | 1117 (73%) | 1553 (76%) | 0.11 |
| Body mass index | 24.1±3.6 | 23.9±3.6 (2010) | 0.04 |
| Coexisting condition |  |  |  |
| Hypertension | 1260 (83%) | 1574 (77%) | <0.0001 |
| Diabetes mellitus | 604 (40%) | 824 (40%) | 0.76 |
| Insulin-treated diabetes | 119 (7.8%) | 176 (8.6%) | 0.41 |
| ESRD (eGFR<30 mL/min/1.73m^2^) not on hemodialysis | 35/1521 (2.3%) | 93/2054 (4.5%) | 0.0003 |
| Hemodialysis | 56 (3.7%) | 141 (6.9%) | <0.0001 |
| Cardiac risk factor |  |  |  |
| Current smoker | 315 (21%) | 430 (21%) | 0.84 |
| Prior Stroke | 168 (11%) | 243 (12%) | 0.45 |
| Heart failure | 101 (6.6%) | 191 (9.3%) | 0.004 |
| Peripheral vascular disease | 142 (9.3%) | 177 (8.6%) | 0.47 |
| Clinical characteristics |  |  |  |
| Clinical presentation |  |  |  |
| Stable coronary artery disease | 1040 (68%) | 1277 (62%) | 0.0002 |
| Acute coronary syndrome | 485 (32%) | 777 (38%) | 0.0002 |
| Unstable angina | 229 (15%) | 299 (15%) | 0.7 |
| Acute myocardial infarction | 256 (17%) | 478 (23%) | <0.0001 |
| STEMI | 213 (14%) | 389 (19%) | <0.0001 |
| NSTEMI | 43 (2.8%) | 89 (4.3%) | 0.02 |
| Target-vessel location |  |  |  |
| Left main coronary artery | 17 (1.1%) | 160 (7.8%) | <0.0001 |
| Left anterior descending coronary artery | 866 (57%) | 1108 (54%) | 0.09 |
| Left circumflex coronary artery | 361 (24%) | 460 (22%) | 0.37 |
| Right coronary artery | 405 (27%) | 614 (30%) | 0.03 |
| Bypass graft | 4 (0.3%) | 17 (0.8%) | 0.02 |
| Complexity of coronary artery disease |  |  |  |
| Number of treated lesions per patient | 1.21±0.48 | 1.43±0.74 | <0.0001 |
| Multi-vessel treatment | 130 (8.5%) | 315 (15%) | <0.0001 |

Values are expressed as mean ± SD or number (%).

ESRD=end stage renal disease; eGFR=estimated glomerular filtration rate; STEMI=ST-segment elevation myocardial infarction; NSTEMI=non-ST-segment elevation myocardial infarction.

We also calculated the GRACE score in ACS patients. Mean GRACE score was 109.4±27.0 in UA, 123.8±32.5 in NSTEMI and 159.6±32.1 in STEMI. We added the GRACE score in Table 1.

**- Statistical analysis: “Sample size calculation of this study was previously described.” The sample size was previously described for the main analysis, and not for this post-hoc analysis. Conversely, the present analysis seems to be underpowered and non-conclusive for its purpose. Please clarify.**

We appreciate the comments.

As reviewer commented, the sample size was previously described for the main analysis, and not for this post-hoc analysis. Therefore, we amended the description in the statistical analysis section as follows.

“Sample size calculation for the main analysis of this study was previously described.” (Page 9, Line 152)

The present analysis is considered to be underpowered and we added following statement in the limitation section.

“Finally, the sample size calculation was conducted for the main analysis, but not for this post-hoc analysis. Therefore, the number of patients enrolled in this study was underpowered, and not large enough to evaluate the low frequency event such as ST, especially in those with ACS.” (Page 15, Line 272)

**- How were the patients on oral anticoagulants managed? Did the duration of DAPT in this subgroup differ from the rest of the population?**

We appreciate the comments.

DAPT duration in patients on oral anticoagulants was the same as the rest of the population. We added the following statement in the method section.

“DAPT duration in patients with oral anticoagulants was the same as the rest of the population.” (Page 6, Line 98)

**- Conclusions: should be extended, and reworded to clarify that most of ACS patients were UA.**

We appreciate the comments.

As reviewer suggested, we amended the conclusion as follows.

“Stopping DAPT at 3-month after CoCr-EES implantation in patients with ACS including 47% of unstable angina was as safe as that in patients with stable CAD.” (Page 3, Line 49, Page 15, Line 275)

**- Please, better define which medications were considered among "strong statins".**

Atorvastatin, pitavastatin and rosuvastatin were considered to be strong statins. We included the following statement in the footnote of Table1.

“Atorvastatin, pitavastatin and rosuvastatin were considered to be strong statins.”

**- Please, diffusely correct typos in the text: i.e., "Statins use" instead of "Stains use" in Table 3.**

We amended "Statins use" instead of "Stains use" in Table 3.

**Reviewer #2:**

In this study the authors aim at comparing the outcome in patients with ACS versus stable CAD undergoing PCI and subsequent double antiplatelet therapy (DAPT) with clopidogrel plus aspirin) for 3 months, followed by single antiplatelet therapy (SAPT) with aspirin for 12 months. Furthermore, the authors compared the clinical outcomes of the STOPDAPT trial with the RESET trial in both ACS and stable CAD patients. The authors concluded that a 3-month DAPT in ACS patients is as safe as in stable CAD patients.
The aim of this study is interesting; however, I have several comments to the authors.

**Major comments:

• The authors stated that, among candidate patients, only those judged eligible by the physician were enrolled. In fact, among 3580 candidate patients, 2054 (far more than the half) were not enrolled. As the authors stated in the limitation section, this leads to a selection bias, that should be more clearly explained by the authors in discussing the results. Furthermore, as in a previous paper of the same trial (DOI 10.1007/s12928-015-0366-9), the main characteristics of not enrolled patients should be presented (also in appendix) or, at least, cited.**

We appreciate the comments.

As reviewer suggested, we presented the patient characteristics of enrolled and non-enrolled patients in the supplemental table 1. Patients with ACS, STEMI and NSTEMI patients were more included in the non-enrolled group than in the enrolled group. We included following statement in the results and limitation section.

“Baseline characteristics were significantly different in several aspects between the enrolled and non-enrolled patients (S1 Table 1). AMI presentation were more prevalent in the non-enrolled group, while stable CAD were more often found in the enrolled group.” (Page 9, Line 158)

“First, selection bias toward inclusion of patients with lower ischemic risk should be considered when interpreting the result of this study. Despite the all-comer study design, those ACS patients with large thrombotic burden and/or extensive atherosclerotic burden might well have not been included in this short DAPT trial. Indeed, patients with STEMI and NSTEMI were more often included in the non-enrolled group.” (Page 14, Line 262)

**S1 Table. Patient Characteristics: Enrolled versus Non-enrolled Patients**

|  | Enrolled | Non-enrolled | P Value |
| --- | --- | --- | --- |
|  | N=1525 | N=2054 |  |
| Age –years | 70.0±10.6 | 70.0±11.0 | 0.97 |
| Age >=75 years | 570 (37%) | 776 (38%) | 0.81 |
| Male gender | 1117 (73%) | 1553 (76%) | 0.11 |
| Body mass index | 24.1±3.6 | 23.9±3.6 (2010) | 0.04 |
| Coexisting condition |  |  |  |
| Hypertension | 1260 (83%) | 1574 (77%) | <0.0001 |
| Diabetes mellitus | 604 (40%) | 824 (40%) | 0.76 |
| Insulin-treated diabetes | 119 (7.8%) | 176 (8.6%) | 0.41 |
| ESRD (eGFR<30 mL/min/1.73m^2^) not on hemodialysis | 35/1521 (2.3%) | 93/2054 (4.5%) | 0.0003 |
| Hemodialysis | 56 (3.7%) | 141 (6.9%) | <0.0001 |
| Cardiac risk factor |  |  |  |
| Current smoker | 315 (21%) | 430 (21%) | 0.84 |
| Prior Stroke | 168 (11%) | 243 (12%) | 0.45 |
| Heart failure | 101 (6.6%) | 191 (9.3%) | 0.004 |
| Peripheral vascular disease | 142 (9.3%) | 177 (8.6%) | 0.47 |
| Clinical characteristics |  |  |  |
| Clinical presentation |  |  |  |
| Stable coronary artery disease | 1040 (68%) | 1277 (62%) | 0.0002 |
| Unstable angina | 229 (15%) | 299 (15%) | 0.7 |
| Acute myocardial infarction | 256 (17%) | 478 (23%) | <0.0001 |
| STEMI | 213 (14%) | 389 (19%) | <0.0001 |
| NSTEMI | 43 (2.8%) | 89 (4.3%) | 0.02 |
| Target-vessel location |  |  |  |
| Left main coronary artery | 17 (1.1%) | 160 (7.8%) | <0.0001 |
| Left anterior descending coronary artery | 866 (57%) | 1108 (54%) | 0.09 |
| Left circumflex coronary artery | 361 (24%) | 460 (22%) | 0.37 |
| Right coronary artery | 405 (27%) | 614 (30%) | 0.03 |
| Bypass graft | 4 (0.3%) | 17 (0.8%) | 0.02 |
| Complexity of coronary artery disease |  |  |  |
| Number of treated lesions per patient | 1.21±0.48 | 1.43±0.74 | <0.0001 |
| Multi-vessel treatment | 130 (8.5%) | 315 (15%) | <0.0001 |

Values are expressed as mean ± SD or number (%).

ESRD=end stage renal disease; eGFR=estimated glomerular filtration rate; STEMI=ST-segment elevation myocardial infarction; NSTEMI=non-ST-segment elevation myocardial infarction.

**• Although the eligibility criteria of the RESET were comparable to those of the STOPDAPT, in the former study all patients with previous DES were excluded. Considering that, in RESET study, more patients that in STOPDAPT had previous PCI, a consistent number of patients in this study had a previous BMS implanted. Furthermore, in STOPDAPT only those judged eligible by the physician were enrolled. Importantly the baseline characteristics of the population of the two trials are strongly different (as shown in table s1), suggesting a higher risk profile in RESET population (more diabetics, more end stage CKD, more MVD…). This suggest that many confounders could bias the results and an adjustment through a propensity score should be performed before comparing the outcome of this two populations.**

We appreciate the comments.

As reviewer commented, the baseline characteristics of the population of the two trials are strongly different, suggesting a higher risk profile in RESET population. Many confounders could bias the result. The editor also commented this point and suggested to omit this comparison. Therefore, we omitted the comparison of two different populations from the STOPDAPT and RESET trials.

**• There are significant differences between the ACS and stable CAD group in terms of many important variables (more AF, PAD, previous MI, Previous PCI/CABG, MVD in stable CAD group). Since these large differences between the two arms could be not adequately adjusted by a multivariable analysis, an adjustment throughout a propensity score would be a better option.**

We appreciate the comments.

As reviewer commented, significant differences between the ACS and stable CAD group in terms of many important variables. Therefore, we conducted the adjustment using propensity score matching.

The propensity score was calculated from the 7 risk-adjusting variables. Using the propensity score, patients in the stable CAD group were randomly matched to ACS patients using a greedy matching strategy. In the propensity matched analysis, the cumulative 1-year incidence of the primary endpoint were similar between the ACS and stable CAD groups (2.3% versus 2.1%, P=0.82).

We added the results of propensity matched analysis in supplemental table2, supplemental table 3 and Figure 6.

**S2 Table. Patient Characteristics in Propensity Matched Cohort**

|  | ACS | Stable CAD | P Value |
| --- | --- | --- | --- |
|  | 487 patients | 487 patients |  |
| **Clinical characteristics** |  |  |  |
| Age – years | 69.0±12.3 | 69.9±10.1 | 0.22 |
| Age >=75 years * | 176 (36%) | 173 (36%) | 0.84 |
| Men | 361 (74%) | 352 (72%) | 0.51 |
| Body mass index | 24.0±3.6 | 24.3±3.5 | 0.13 |
| Hypertension | 406 (83%) | 389 (80%) | 0.16 |
| Diabetes mellitus * | 162 (33%) | 165 (34%) | 0.84 |
| Dyslipidemia | 386 (79%) | 420 (86%) | 0.004 |
| Hemodialysis * | 8 (1.6%) | 8 (1.6%) | 1.0 |
| Atrial fibrillation * | 39 (8.0%) | 39 (8.0%) | 1.0 |
| Anemia (Hemoglobin <11.0 g/dL) | 76 (16%) | 57 (12%) | 0.08 |
| Current smoker | 142 (29%) | 77 (16%) | <0.0001 |
| Prior myocardial infarction * | 38 (7.8%) | 37 (7.6%) | 0.9 |
| Prior Stroke | 49 (10%) | 44 (9.0%) | 0.57 |
| Heart failure | 58 (12%) | 45 (9.2%) | 0.18 |
| Peripheral vascular disease * | 19 (3.9%) | 20 (4.1%) | 0.87 |
| Prior percutaneous coronary intervention | 80 (16%) | 152 (31%) | <0.0001 |
| Multivessel disease | 141 (29%) | 197 (40%) | 0.0002 |
| Medications |  |  |  |
| Aspirin | 487 (100%) | 487 (100%) | - |
| Thienopyridines | 487 (100%) | 486 (99.8%) | 0.24 |
| Statins * | 438 (90%) | 439 (90%) | 0.91 |
| B-blockers | 256 (53%) | 152 (31%) | <0.0001 |
| ACE-I/ARB | 334 (69%) | 264 (54%) | 0.0001 |
| Anti-coaglants | 45 (9.2%) | 38 (7.8%) | 0.42 |
| **Lesion and procedural characteristics** |  |  |  |
| No. of treated lesions per patient | 1.14±0.42 | 1.26±0.56 | <0.0001 |
| No. of stents used per patient | 1.27±0.56 | 1.4±0.67 | 0.0008 |
| Total stent length per patient - mm | 30.7±18.0 | 33.2±21.2 | 0.048 |

**S3 Table. Clinical Outcomes at 12 months in Propensity Matched Cohort**

|  | No. of patients with at least one event | | P Value |
| --- | --- | --- | --- |
|  | (Cumulative incidence) | |  |
|  | ACS | Stable CAD |  |
|  | N=487 | N=487 |  |
| Primary Endpoint | 11 (2.3%) | 10 (2.1%) | 0.82 |
| Death |  |  |  |
| All-cause | 5 (1.0%) | 5 (1.0%) | 0.99 |
| Cardiovascular death | 2 (0.4%) | 3 (0.6%) | 0.66 |
| Myocardial infarction | 1 (0.2%) | 2 (0.4%) | 0.56 |
| Stroke | 4 (0.8%) | 3 (0.6%) | 0.7 |
| Bleeding |  |  |  |
| TIMI minor/major | 6 (1.2%) | 2 (0.4%) | 0.15 |
| GUSTO moderate/severe | 4 (0.8%) | 2 (0.4%) | 0.41 |
| Stent thrombosis |  |  |  |
| Possible | 0 (0%) | 2 (0.4%) | 0.16 |
| Probable | 0 (0%) | 0 (0%) |  |
| Definite | 0 (0%) | 0 (0%) |  |
| Cardiovascular death, MI, stroke or definite ST | 7 (1.4%) | 8 (1.7%) | 0.8 |
| Target-lesion revascularization | 8 (1.7%) | 6 (1.2%) | 0.58 |
| Target-vessel revascularization | 15 (3.1%) | 14 (2.9%) | 0.85 |
| Any coronary revascularization | 30 (6.2%) | 29 (6.0%) | 0.9 |

Figure 6.

**• The fact that in stable CAD 6 possible stent thrombosis occurred against 0 in ACS group clearly reflect a difference among the two populations. The same is for the difference, in terms death ((particularly non-cardiac death), between ACS and CAD at the landmark analysis. It is difficult to believe that that chronic CAD patients had a worse outcome.**

We appreciate the comments.

As reviewer commented, the fact that in stable CAD 6 possible stent thrombosis occurred against 0 in ACS group clearly reflect a difference among the two populations. In the propensity matched analysis, cumulative incidence of the primary endpoint, all-cause death and stent thrombosis were similar between the 2 groups (supplemental table 2).

**• If the aim of the study is to demonstrate that, after an ACS, a 3-month DAPT after CoCr-EES implantation in patients with ACS is as safe as (or not less safe than) in stable CAD patients, a non-inferiority analysis should be planned. In fact, the non-significance at a superiority analysis cannot be used to demonstrate that there is not any significant difference between the two arms nor that an arm is not inferior to a second arm. This is particularly true for the endpoint for which the study is underpowered (e.g. stent thrombosis).**

We appreciate the comments.

The adjusted risk of ACS relative to stable CAD for the primary endpoint was HR 0.94, 95%CI 0.46-1.92. The large 95% CI with neutral point estimate indicated that the study was underpowered for the non-inferiority analysis even for the primary endpoint.

**• It should be discussed that, for this study, the external validity to non-Asian patients is limited firstly because the study was conducted only in Japanese centres, secondly because the main P2Y12 inhibitor used was Clopidogrel (whereas ticagrelor or prasugrel should be generally preferred, especially in non-Asian patients)**

We appreciate the comments.

As reviewer commented, the external validity to non-Asian patients is limited. Therefore, we added the following statement in the limitation section.

“Third, patients received clopidogrel as a P2Y12 receptor blocker even in patients with ACS. Prasugrel or ticagrelor were not available in the enrollment period between 2012 and 2013 in Japan. Prasugrel or ticagrelor are generally preferred in ACS patients in the current clinical practice, especially in non-Asian patients. Fourth, as this study was conducted only in Japanese centers, the external validity to non-Asian patients would be limited.” (Page 15, Line 268)

**Minor comments:
• In line 268, why do the authors use the plural form for STOPDAPT-2 ACS trial?**

We amended“STOPDAPT-2 ACS trials” to“STOPDAPT-2 ACS trial”. **• I think that hyphens are sometimes incorrectly used. For example, in conclusion section, “Stopping DAPT at 3-month” should be replaced with “Stopping DAPT at 3 months”.**

We amended“Stopping DAPT at 3-month” to“Stopping DAPT at 3 months”.

**Reviewer #3:**

**The manuscript addresses an interesting topic. The data are of certain interest and the employed methods are sound. The results might be very useful for future researchers. Some comments follow.**

**1. Data are not fully available. This is not in line with the journal's guidelines. Please, update the data and the code used to obtain parameters estimates. This would ensure the reproducibility of the results and allow the reviewers to check for the appropriateness of the methods.**

Thank you very much for your comment.

Because this was conducted as clinical trial and informed consent was obtained individually. Because the Japanese ethics guideline requested the investigator to obtain permission from subjects and approval from local ethics committee to provide data outside Japan, we cannot provide or disclose the data to public. However, we are happy to provide the analyses codes and the output of analyses to the reviewers if editors and reviewers considered it necessary to review our manuscript.

**2. My main doubts concern some aspects of the modelling.**

**2a. Firstly, the authors must check for heterogeneity between centers. A major, and quite strong, assumption in the modelling is that patients belonging to different centers are homogeneous, i.e. no differences arise between multiple centers. Please, provide an extension of the modelling that takes into account for the presence of heterogeneity between centers. A random effects model may be an option.**

We appreciate the comments.

As reviewer commented, patients belonging to different centers are homogeneous. Therefore, we included center as a risk adjusting variable and conducted multivariate analysis with a random effect of center.

Adjusted HR for the primary endpoint was similar with the current model. We amended the Table 3 as follows and added the following statement in the method section.

“The presentation (ACS or stable CAD), the 7 risk-adjusting variables and random effect of center were simultaneously included in the Cox proportional hazard model.” (Page 8, Line 145)

**Table 3. Unadjusted and adjusted risks of the ACS versus non-ACS and the effects of the risk-adjusting variables for the primary endpoint**

|  | Unadjusted | P value | Adjusted | P value |
| --- | --- | --- | --- | --- |
|  | HR (95%CI) |  | HR (95%CI) |  |
| ACS | 0.75 (0.36-1.46) | 0.41 | 0.94 (0.46-1.92) | 0.87 |
| Age >=75 years | 2.53 (1.38-4.76) | 0.003 | 2.1 (1.11-3.94) | 0.02 |
| Diabetes | 0.76 (0.39-1.42) | 0.4 | 0.71 (0.37-1.36) | 0.3 |
| Hemodialysis | 2.04 (0.49-5.62) | 0.28 | 1.62 (0.49-5.36) | 0.43 |
| Atrial fibrillation | 2.53 (1.18-4.97) | 0.02 | 2.03 (0.98-4.19) | 0.06 |
| Prior myocardial infarction | 1.48 (0.69-2.91) | 0.29 | 1.54 (0.74-3.19) | 0.24 |
| Peripheral vascular disease | 3.14 (1.46-6.15) | 0.005 | 2.54 (1.23-5.28) | 0.01 |
| Statins use | 0.78 (0.4-1.67) | 0.5 | 0.9 (0.43-1.88) | 0.78 |

**2b. The use of Cox survival modelling is sound. Nevertheless, several assumptions must be fulfilled to ensure a proper statistical inference. It is desirable to determine whether a fitted Cox regression model adequately describes the data. I will briefly consider three kinds of diagnostics: for violation of the assumption of proportional hazards; for influential data; and for nonlinearity in the relationship between the log hazard and the covariates. Please, provide evidence that model assumptions are fulfilled or, if thery not, modify the model specification accordingly.**

We appreciate your clarification.

We checked the proportional hazard assumptions for the risk-adjusting variables were assessed on the plots of log (time) versus log [-log (survival)] stratified by the variable. The assumptions were verified to be acceptable for all the variables. Therefore, we added the following statement in the method section.

“Proportional hazard assumptions for the risk-adjusting variables were assessed on the plots of log (time) versus log [-log (survival)] stratified by the variable. The assumptions were verified to be acceptable for all the variables.” (Page 8, Line 147)

**2c. As minor points: please explain the difference between adjusted and non-adjusted coefficients; furthermore, please consider a model selection procedure (e.g. LASSO) to identify the relevant variables.**

Thank you very much for clarification.

We used the clinical relevant factors for the adjustment. Considering the small number of events, we used the following 7 factors for adjustment. We did not employ any model selection procedure. We consider this method is the standard one for clinical research in this field.

**Reviewer #4:**

**- In consideration of the relevant clinical and angiographic differences between the two compared groups, you have to perform a propensity pair-matched analysis, to increase accuracy of your results. Your current adjusted analysis is very limited and you have the number to perform a better analysis.**

We appreciate the comments.

As reviewer suggested, we conducted the adjustment using propensity matched analysis.

The propensity score was calculated from the 7 risk-adjusting variables. Using the propensity score, patients in the stable CAD group were randomly matched to ACS patients using a greedy matching strategy. In the propensity matched analysis, the cumulative 1-year incidence of the primary endpoint was similar between the ACS and stable CAD groups (2.3% versus 2.1%, P=0.82).

We added the results of propensity matched analysis in supplemental table 2, supplemental table 3 and Figure 6.

**S2 Table. Patient Characteristics in Propensity Matched Cohort**

|  | ACS | Stable CAD | P Value |
| --- | --- | --- | --- |
|  | 487 patients | 487 patients |  |
| **Clinical characteristics** |  |  |  |
| Age – years | 69.0±12.3 | 69.9±10.1 | 0.22 |
| Age >=75 years * | 176 (36%) | 173 (36%) | 0.84 |
| Men | 361 (74%) | 352 (72%) | 0.51 |
| Body mass index | 24.0±3.6 | 24.3±3.5 | 0.13 |
| Hypertension | 406 (83%) | 389 (80%) | 0.16 |
| Diabetes mellitus * | 162 (33%) | 165 (34%) | 0.84 |
| Dyslipidemia | 386 (79%) | 420 (86%) | 0.004 |
| Hemodialysis * | 8 (1.6%) | 8 (1.6%) | 1.0 |
| Atrial fibrillation * | 39 (8.0%) | 39 (8.0%) | 1.0 |
| Anemia (Hemoglobin <11.0 g/dL) | 76 (16%) | 57 (12%) | 0.08 |
| Current smoker | 142 (29%) | 77 (16%) | <0.0001 |
| Prior myocardial infarction * | 38 (7.8%) | 37 (7.6%) | 0.9 |
| Prior Stroke | 49 (10%) | 44 (9.0%) | 0.57 |
| Heart failure | 58 (12%) | 45 (9.2%) | 0.18 |
| Peripheral vascular disease * | 19 (3.9%) | 20 (4.1%) | 0.87 |
| Prior percutaneous coronary intervention | 80 (16%) | 152 (31%) | <0.0001 |
| Multivessel disease | 141 (29%) | 197 (40%) | 0.0002 |
| Medications |  |  |  |
| Aspirin | 487 (100%) | 487 (100%) | - |
| Thienopyridines | 487 (100%) | 486 (99.8%) | 0.24 |
| Statins * | 438 (90%) | 439 (90%) | 0.91 |
| B-blockers | 256 (53%) | 152 (31%) | <0.0001 |
| ACE-I/ARB | 334 (69%) | 264 (54%) | 0.0001 |
| Anti-coaglants | 45 (9.2%) | 38 (7.8%) | 0.42 |
| **Lesion and procedural characteristics** |  |  |  |
| No. of treated lesions per patient | 1.14±0.42 | 1.26±0.56 | <0.0001 |
| No. of stents used per patient | 1.27±0.56 | 1.4±0.67 | 0.0008 |
| Total stent length per patient - mm | 30.7±18.0 | 33.2±21.2 | 0.048 |

**S3 Table. Clinical Outcomes at 12 months in Propensity Matched Cohort**

|  | No. of patients with at least one event | | P Value |
| --- | --- | --- | --- |
|  | (Cumulative incidence) | |  |
|  | ACS | Stable CAD |  |
|  | N=487 | N=487 |  |
| Primary Endpoint | 11 (2.3%) | 10 (2.1%) | 0.82 |
| Death |  |  |  |
| All-cause | 5 (1.0%) | 5 (1.0%) | 0.99 |
| Cardiovascular death | 2 (0.4%) | 3 (0.6%) | 0.66 |
| Myocardial infarction | 1 (0.2%) | 2 (0.4%) | 0.56 |
| Stroke | 4 (0.8%) | 3 (0.6%) | 0.7 |
| Bleeding |  |  |  |
| TIMI minor/major | 6 (1.2%) | 2 (0.4%) | 0.15 |
| GUSTO moderate/severe | 4 (0.8%) | 2 (0.4%) | 0.41 |
| Stent thrombosis |  |  |  |
| Possible | 0 (0%) | 2 (0.4%) | 0.16 |
| Probable | 0 (0%) | 0 (0%) |  |
| Definite | 0 (0%) | 0 (0%) |  |
| Cardiovascular death, MI, stroke or definite ST | 7 (1.4%) | 8 (1.7%) | 0.8 |
| Target-lesion revascularization | 8 (1.7%) | 6 (1.2%) | 0.58 |
| Target-vessel revascularization | 15 (3.1%) | 14 (2.9%) | 0.85 |
| Any coronary revascularization | 30 (6.2%) | 29 (6.0%) | 0.9 |

Figure 6.

**- Events tended to be higher in the stable CAD groups, please provides analysis to better understand this greater risk.**

We appreciate the comments. As reviewer commented, events tended to be higher in the stable CAD group. Baseline characteristics between the two groups were significantly different in many factors, suggesting a higher risk profile in stable CAD population. Therefore, we conducted the adjustment using propensity matched analysis.

In the propensity matched analysis, the cumulative 1-year incidence of the primary endpoint were similar between the ACS and stable CAD groups (2.3% versus 2.1%, P=0.82).

**- You have to better explain what are the scientific and practical implications of your data and findings. Try to reinforce the overall meaning of your data.**

We appreciate the comments. As reviewer commented, the scientific and practical implication of this study should better be explained. We amended the following statement in the discussion section.

“In the present post-hoc analysis of the STOPDAPT trial demonstrated that 3-month DAPT after CoCr-EES implantation was associated with similarly low event rate for the primary endpoint without any definite ST events in both ACS and stable CAD patients, although 47% of ACS patients had troponin-negative UA at low-risk of event recurrence. Furthermore, the STOPDAPT trial had mostly enrolled stable patients after PCI, which might be one of the reasons for the low event rate even in ACS patients. Therefore, 3-month DAPT could be an option in selected patients with ACS in the new-generation DES era.”(Page 14, Line 250)

**Reviewer #5:**

**It is unclear how events occurred more often in the Stable CAD rather than in the ACS arm. ACS patients including NSTEMI and STEMI patients have a significantly higher risk of ischemic and bleeding complication compared to stable patients. This point should be thoroughly evaluated as it hampers to draft conclusion from this data. In fact ACS at univariate analysis seems tendentially protective from adverse events, which tend to lessen after adjustment. Ultimately it is not clear to this reviewer the comparison between ACS and SCAD for clinical events considering that due to more conservative patient selection in the ACS arm (see Table 1). STOPDAPT as a single arm study cannot either give information regarding the impact of different terms of DAPT duration.**

We appreciate the comments.

As reviewer commented, ACS patients including NSTEMI and STEMI patients have a significantly higher risk of ischemic and bleeding complication compared to stable patients. However, event rates in this study tended to be higher in stable CAD patients than in ACS patients. These results might be related to the high prevalence of patients with unstable angina in the ACS group. Therefore, we amended the conclusion as follows.

“Stopping DAPT at 3-month after CoCr-EES implantation in patients with ACS including 47% of unstable angina was as safe as that in patients with stable CAD.” (Page 3, Line 49, Page 15, Line 276)

Furthermore, we conducted the adjustment using propensity score matching. In the propensity matched analysis, the cumulative 1-year incidence of the primary endpoint were similar between the ACS and stable CAD groups (2.3% versus 2.1%, P=0.82).

We added the results of propensity matched analysis in supplemental table2, supplemental table 3 and Figure 6.

**S2 Table. Patient Characteristics in Propensity Matched Cohort**

|  | ACS | Stable CAD | P Value |
| --- | --- | --- | --- |
|  | 487 patients | 487 patients |  |
| **Clinical characteristics** |  |  |  |
| Age – years | 69.0±12.3 | 69.9±10.1 | 0.22 |
| Age >=75 years * | 176 (36%) | 173 (36%) | 0.84 |
| Men | 361 (74%) | 352 (72%) | 0.51 |
| Body mass index | 24.0±3.6 | 24.3±3.5 | 0.13 |
| Hypertension | 406 (83%) | 389 (80%) | 0.16 |
| Diabetes mellitus * | 162 (33%) | 165 (34%) | 0.84 |
| Dyslipidemia | 386 (79%) | 420 (86%) | 0.004 |
| Hemodialysis * | 8 (1.6%) | 8 (1.6%) | 1.0 |
| Atrial fibrillation * | 39 (8.0%) | 39 (8.0%) | 1.0 |
| Anemia (Hemoglobin <11.0 g/dL) | 76 (16%) | 57 (12%) | 0.08 |
| Current smoker | 142 (29%) | 77 (16%) | <0.0001 |
| Prior myocardial infarction * | 38 (7.8%) | 37 (7.6%) | 0.9 |
| Prior Stroke | 49 (10%) | 44 (9.0%) | 0.57 |
| Heart failure | 58 (12%) | 45 (9.2%) | 0.18 |
| Peripheral vascular disease * | 19 (3.9%) | 20 (4.1%) | 0.87 |
| Prior percutaneous coronary intervention | 80 (16%) | 152 (31%) | <0.0001 |
| Multivessel disease | 141 (29%) | 197 (40%) | 0.0002 |
| Medications |  |  |  |
| Aspirin | 487 (100%) | 487 (100%) | - |
| Thienopyridines | 487 (100%) | 486 (99.8%) | 0.24 |
| Statins * | 438 (90%) | 439 (90%) | 0.91 |
| B-blockers | 256 (53%) | 152 (31%) | <0.0001 |
| ACE-I/ARB | 334 (69%) | 264 (54%) | 0.0001 |
| Anti-coaglants | 45 (9.2%) | 38 (7.8%) | 0.42 |
| **Lesion and procedural characteristics** |  |  |  |
| No. of treated lesions per patient | 1.14±0.42 | 1.26±0.56 | <0.0001 |
| No. of stents used per patient | 1.27±0.56 | 1.4±0.67 | 0.0008 |
| Total stent length per patient - mm | 30.7±18.0 | 33.2±21.2 | 0.048 |

**S3 Table. Clinical Outcomes at 12 months in Propensity Matched Cohort**

|  | No. of patients with at least one event | | P Value |
| --- | --- | --- | --- |
|  | (Cumulative incidence) | |  |
|  | ACS | Stable CAD |  |
|  | N=487 | N=487 |  |
| Primary Endpoint | 11 (2.3%) | 10 (2.1%) | 0.82 |
| Death |  |  |  |
| All-cause | 5 (1.0%) | 5 (1.0%) | 0.99 |
| Cardiovascular death | 2 (0.4%) | 3 (0.6%) | 0.66 |
| Myocardial infarction | 1 (0.2%) | 2 (0.4%) | 0.56 |
| Stroke | 4 (0.8%) | 3 (0.6%) | 0.7 |
| Bleeding |  |  |  |
| TIMI minor/major | 6 (1.2%) | 2 (0.4%) | 0.15 |
| GUSTO moderate/severe | 4 (0.8%) | 2 (0.4%) | 0.41 |
| Stent thrombosis |  |  |  |
| Possible | 0 (0%) | 2 (0.4%) | 0.16 |
| Probable | 0 (0%) | 0 (0%) |  |
| Definite | 0 (0%) | 0 (0%) |  |
| Cardiovascular death, MI, stroke or definite ST | 7 (1.4%) | 8 (1.7%) | 0.8 |
| Target-lesion revascularization | 8 (1.7%) | 6 (1.2%) | 0.58 |
| Target-vessel revascularization | 15 (3.1%) | 14 (2.9%) | 0.85 |
| Any coronary revascularization | 30 (6.2%) | 29 (6.0%) | 0.9 |

Figure 6.
