# Supplementary material for: One-year clinical outcomes of patients with versus without acute coronary syndrome with 3-month duration of dual antiplatelet therapy after everolimus-eluting stent implantation
Source: PLoS One. 2020 Mar 25;15(3):e0227612. doi: 10.1371/journal.pone.0227612 (PMC7094877; doi:10.1371/journal.pone.0227612)
Supplement: Attachment — Submitted filename: Responses to reviewers STOPDAPT ACS revise2 final version.docx [file pone.0227612.s007.docx]

**Responses to the reviewer’s comments**

We appreciate the kind editing and comments from the editor and the reviewers.

**Reviewer #3:**

**One point needs still to be addressed. Influential observations and nonlinearities in the Cox model should be analyzed. Yhe martingale residuals may be plotted against covariates to detect nonlinearity, and may also be used to form component-plus-residual (or partial-residual) plots, again in the manner of linear and generalized linear models. A matrix of estimated changes in the regression coefficients upon deleting each observation in turn may be used to detect influential observations.**

Thank you very much for your comment.

As reviewer commented, influential observations and nonlinearities in the Cox model could be analyzed. Proportional hazard assumptions for the risk-adjusting variables were assessed on the plots of log (time) versus log [-log (survival)] stratified by the variable. The assumptions were verified to be acceptable for all the variables. We thus here show the plots for age, atrial fibrillation (AF), and peripheral vascular disease (PVD) because these variables had smaller p-values.

The main comparison of this study is ACS versus non-ACS patients, and thus other covariates are used for adjustment in the multivariate models and not relevant to the main results of this study. Therefore, the detailed inspections for residuals or linearity are not considered relevant to the manuscript and they do not affect on our results. However, we are happy to conduct these analyses if the editors and reviewers considered it necessary to accept our manuscript.
